# PARP1-DOT1L transcription axis drives acquired resistance to PARP inhibitor in ovarian cancer

**DOI:** 10.1186/s12943-024-02025-8

**Published:** 2024-05-22

**Authors:** Chaohua Liu, Jiana Li, Fei Xu, Lihua Chen, Mengdong Ni, Jiangchun Wu, Haiyun Zhao, Yangjun Wu, Jiajia Li, Xiaohua Wu, Xiaojun Chen

**Affiliations:** 1 Department of Gynecologic Oncology, Fudan University Shanghai Cancer Center, Shanghai, China; 2 Cancer Institute, Fudan University Shanghai Cancer Center, Shanghai, China; 3 Department of Oncology, Shanghai Medical College, Fudan University, Shanghai, China

**Keywords:** DOT1L, PARP1, PARPi resistance, Ovarian cancer

## Abstract

**Background:**

Poly (ADP-ribose) polymerase inhibitor (PARPi) resistance poses a significant challenge in ovarian carcinoma (OC). While the role of DOT1L in cancer and chemoresistance is acknowledged, its specific role in PARPi resistance remains unclear. This study aims to elucidate the molecular mechanism of DOT1L in PARPi resistance in OC patients.

**Methods:**

This study analyzed the expression of DOT1L in PARPi-resistant cell lines compared to sensitive ones and correlated it with clinical outcomes in OC patients. Comprehensive in vitro and in vivo functional experiments were conducted using cellular and mouse models. Molecular investigations, including RNA sequencing, chromatin immunoprecipitation (ChIP) and Cleavage Under Targets and Tagmentation (CUT&Tag) assays, were employed to unravel the molecular mechanisms of DOT1L-mediated PARPi resistance.

**Results:**

Our investigation revealed a robust correlation between DOT1L expression and clinical PARPi resistance in non-BRCA mutated OC cells. Upregulated DOT1L expression in PARPi-resistant tissues was associated with diminished survival in OC patients. Mechanistically, we identified that PARP1 directly binds to the DOT1L gene promoter, promoting transcription independently of its enzyme activity. PARP1 trapping induced by PARPi treatment amplified this binding, enhancing DOT1L transcription and contributing to drug resistance. Sequencing analysis revealed that DOT1L plays a crucial role in the transcriptional regulation of PLCG2 and ABCB1 via H3K79me2. This established the PARP1-DOT1L-PLCG2/ABCB1 axis as a key contributor to PARPi resistance. Furthermore, we discovered that combining a DOT1L inhibitor with PARPi demonstrated a synergistic effect in both cell line-derived xenograft mouse models (CDXs) and patient-derived organoids (PDOs).

**Conclusions:**

Our results demonstrate that DOT1L is an independent prognostic marker for OC patients. The PARP1-DOT1L/H3K79me2-PLCG2/ABCB1 axis is identified as a pivotal contributor to PARPi resistance. Targeted inhibition of DOT1L emerges as a promising therapeutic strategy for enhancing PARPi treatment outcomes in OC patients.

**Supplementary Information:**

The online version contains supplementary material available at 10.1186/s12943-024-02025-8.

## Background

Ovarian carcinoma (OC) is the most lethal gynecological malignancy worldwide, accounting for 4% of all cancers in women [1–3]. The traditional standard of treatment for patients with advanced-stage OC is cytoreductive surgery with platinum and paclitaxel-based neoadjuvant or adjuvant chemotherapy [4–6]. Despite advances in surgical and chemotherapeutic treatment, the 5-year survival rate of advanced OC patients is still 35–40%, largely due to the development of cisplatin resistance [7, 8]. Recently, PARP inhibitors (PARPi), including Olaparib and Niraparib, have been approved for the maintenance therapy of advanced ovarian cancer following first-line chemotherapy [9, 10]. Although PARPi maintenance therapy significantly extends progression-free survival (PFS) in ovarian cancer patients, an increasing number of patients develop primary or acquired resistance to PARPi, limiting its long-term efficacy [11, 12]. Therefore, it is necessary to investigate the mechanisms of PARPi resistance and discover novel combination therapies for PARPi-resistant OC in order to improve patient survival.

The mechanisms of PARPi resistance in clinical settings include restoration of HR repair, re-establishment of replication fork stability, protection of replication fork stability, restoration of PARP1 signaling, and increased drug efflux [11–13]. Drug efflux is dependent on ATP-binding cassette (ABC) transporters, including ABCB1 (P-glycoprotein, MDR1) [14, 15]. In addition, the dysregulation of stemness and metastasis-related genes also plays an important role in drug resistance. The PLCG2 (Phospholipase C, gamma 2)-high cancer phenotype has stem-like, pro-metastatic features that contribute further to acquired therapeutic resistance [16, 17]. Therefore, the disorderly gene regulation profiles among these genes are vital to the development of PARPi resistance.

Epigenetic processes can mediate resistance to targeted therapies and represent novel therapeutic targets, especially in tumors lacking clear genetic mechanisms of resistance [18, 19]. Thus, epigenetic regulators have served as potential targets for cancer therapy, and several drugs have been approved or are currently undergoing clinical trials by the FDA, including inhibitors against DOT1L/KMT4 [20–22]. DOT1L is a non-SET domain methyltransferase that catalyzes H3K79 methylation, and participates in DNA repair, transcription and recombination. In leukemia and solid tumors, DOT1L has been shown to have the cellular function of a tumor-promoter [23–26]. Notably, DOT1L is highly expressed and plays a vital role in the malignant progression of a variety of cancers [23, 27, 28], including OC [29]. Consequently, DOT1L represents a promising drug target.

Furthermore, DOT1L also participates in the regulation of chemotherapy resistance in several tumors [21, 30]. For instance, Liu et al. observed that C/EBPβ enhances platinum resistance in ovarian cancer cells by reprogramming DOT1L/H3K79 methylation to maintain an open chromatin state [31], thereby augmenting the cisplatin resistance of tumor cells. Another recent study demonstrated that co-treatment with DOT1L and menin pharmacological inhibitors exerts an additive effect on growth inhibition in chemotherapy-sensitive and refractory OC cells [32]. However, the role of DOT1L in the development of PARPi resistance in ovarian cancer remains unclear. The optimal efficacy and specific mechanisms of combinational targeting of this epigenetic modifier and the PARPi therapy approach still need to be explored.

In the current study, we identified that DOT1L is one of the most promising candidate targets for PARPi resistance via RNA-seq performed in OC cells and their OlaR counterparts. Through clinicopathologic and survival analysis, as well as in vitro studies of OC cells, we demonstrate the connection of DOT1L to the development of PARPi resistance and further elucidate the potential molecular mechanisms underlying PARPi resistance. Furthermore, we investigated potential treatment strategies for PARPi-resistant OC and demonstrated that genetic and pharmacological inhibition of DOT1L could be employed to overcome PARPi resistance. This suggests that DOT1L may be a valuable novel therapeutic agent against PARPi-resistant ovarian cancer.

## Methods

### Cell culture and establishment of stable cell lines

The human ovarian cancer cell lines used in this paper, SKOV-3, OVCAR8, OVCAR3, OVCA433, HEY, TOV-112D, and Hey-A8 were obtained from ATCC and cultured in Dulbecco’s Modified Eagle Medium (DMEM, Gibco) with 10% fetal bovine serum (FBS, Gibco) and penicillin (100 U/mL)/streptomycin (0.1 mg/mL) (15140-122, Gibco). For the establishment of stable cell lines, the lentivirus expression plasmids were co-transfected with pxPAX2 and pMD2.G into HEK293T cells. Cells were placed in fresh DMEM for 8 h following transfection. The culture media containing the lentiviral particles were then harvested after 48–60 h of incubation. Lentivirus infection was performed by incubating cells with the virus-containing medium with polybrene for 24 h. Stable cells were then selected on puromycin (1–2 µg/ml). The indicated shRNA sequences are listed in Supplementary Table S1.

### Determination of 50% inhibitory concentration

To determine 50% inhibitory concentration (IC50) values of Olaparib (AZD2281; TargetMol, USA), Niraparib (MK-4827; TargetMol, USA), Veliparib (T2591; TargetMol, USA), and Talazoparib (T6523, TargetMol, USA), we measured the cell proliferation rate using Cell Counting Kit-8 (CCK-8) (YEASEN, Shanghai, China). IC50 values were analyzed using GraphPad Prism Version 8.0.

### Drug synergy assay

DOT1L inhibitor SGC0946 and PARP inhibitors Olaparib, Niraparib, Veliparib, and Talazoparib were all purchased from TargetMol. For drug synergy studies, ovarian cancer cells (4000/well) were seeded in 96-well plates for overnight incubation and treated with different doses of inhibitors for 5 days. Cell viability was evaluated by measuring the 450 nm absorbance with the Cell Counting Kit-8 (CCK-8) (YEASEN, Shanghai, China). Each concentration was tested in triplicate. The IC50 value was calculated and performed in GraphPad Prism v8.0. Drug synergistic effects were calculated based on the CompuSyn software or SynergyFinder. CI < 1 indicated synergism, CI = 1 indicated additive effects, and CI > 1 indicated antagonism.

### Cell apoptosis assay

Treated cells were washed with PBS, digested using trypsin, rinsed in PBS, and then resuspended in 1× binding buffer (YEASEN, Shanghai, China). Cells were incubated with fluorescein isothiocyanate (FITC)-Annexin V (5 µL) for 5 min and 7-AAD (10 µL) for 10 min in the dark at 4 °C. The mixture was further analyzed with a BD Flow Cytometer and the FlowJo software.

### Colony formation assay

Cells (2000 cells/well) were plated in 6-well plates as single-cell suspensions, incubated for 24 h, and treated with drugs for 7–14 days. The colonies were fixed with 4% formalin for 15 min and stained with 0.05% crystal violet (Servicebio, Wuhai, China) for 15 min. The number of colonies was counted using the ImageJ 1.52a software.

### Western blot

Cells were lysed with RIPA buffer in the presence of a protease inhibitor cocktail (Roche). The protein concentration was determined by a BCA protein assay kit (Wanleibio, Shenyang, China). Equal amounts of proteins were size fractionated by 6-15% SDS-PAGE. gel and transferred into a polyvinylidene difluoride (PVDF) membrane in a wet electron transfer device. 5% skimmed milk in Tris-buffered saline (TBS) containing 0.05% Tween 20 was used to block the membrane for 2 h at room temperature. The blots were incubated with specific antibodies against human primary antibodies, and the signals were detected using horseradish peroxidase-linked anti-mouse or anti-rabbit conjugates as appropriate and visualized using an ECL detection system (GE Healthcare).

### Establishment of Olaparib-resistant model

Ovarian cancer cell lines were subjected to a gradual increase in the concentration of Olaparib (from 0.5 to 20 µM) to allow for the development of acquired resistance. Cells with acquired resistance to Olaparib (designated as OlaR) were developed after 3–4 months in drug media. The established Olaparib resistance models were maintained in culture medium with low-concentration Olaparib, and dosing was temporarily ceased prior to conducting experiments.

### Antibodies

The antibodies in this study included: H3K79me2 (ab3594, Abcam), Histone H3 (ab1971, Abcam), DOT1L (A300-953 A, Bethyl; sc-390,879, Santa Cruz), PARP1 (13371-1-AP, Proteintech), Flag (F1804, Sigma), P glycoprotein (22336-1-AP, Proteintech), PLCG2 (PTM-6859, PTMBIO), β-tubulin (10068-1-AP, Proteintech), and γ-H2AX (#2577, Cell Signaling Technology).

### PARP1‑DNA trapping assay

1 × 10^6^ cells were treated with 10µM Olaparib for 12 h before being harvested for fractionation. The Subcellular Protein Fractionation Kit for Cultured Cells (ThermoFisher Scientific #78,840) was used for cellular fractionation according to the manufacturer’s instructions. Nuclear-soluble and chromatin-bound fractions were then subjected to immunoblotting.

### CUT&Tag and data analysis

CUT&Tag was performed as previously described [33]. Briefly, 1 × 10^5^ cells were harvested in NE buffer (20 mM HEPES-KOH, pH 7.5, 0.5 mM Spermidine, 10 mM KCl, 0.1% TritonX-100, 10% Glycerol, 1 mM PMSF) and iced for 10 min. ConA beads were pre-washed and resuspended by binding buffer (20 mM HEPES-KOH, pH 7.5, 10 mM KCl, 1 mM CaCl2, 1 mM MnCl2). 10 µl beads were added to each sample and incubated at room temperature for 10 min. The beads were washed with washing buffer (20 mM HEPES-KOH, pH 7.5, 0.5 mM spermidine, 150 mM NaCl, 0.1% BSA) and resuspended in blocking buffer (20 mM HEPES-KOH, pH 7.5, 0.5 mM spermidine, 150 mM NaCl, 0.1% BSA, 2 mM EDTA) at room temperature for 5 min. Primary antibodies (Rabbit monoclonal anti-Histone H3K79me2, CST, 5427 S) were added by 1:100 dilution and incubated at room temperature for 2 h. After being washed with washing buffer, secondary antibodies were added by 1:100 dilution and incubated at room temperature for 30 min. 1.2 µl PA-Tn5 transposomes were added to each sample and incubated at room temperature for 30 min. Beads were resuspended in 30 µl washing buffer with 10 mM MgCl2 and incubated at 37 °C for 1 h. Reactions were stopped by adding 5.5 µl stop buffer (2.25 µL of 0.5 M EDTA, 2.75 µL of 10% SDS and 0.5 µL of 20 mg/ ml Proteinase K) and incubated at 55 °C for 30 min, and then 70 °C for 20 min to inactivate Proteinase K. 0.9X of VAHTS DNA clean beads (VAHTS, Cat. #N411-03) were added to each sample to extract the tagmentated DNA. DNA was purified using phenol-chloroform-isoamyl alcohol extraction and ethanol precipitation. To amplify libraries, 21 µL DNA was mixed with 2 µL of a universal i5 and a uniquely barcoded i7 primer. A volume of 25 µL NEBNext HiFi 2× PCR Master Mix was added and mixed. The sample was placed in a Thermo cycler with a heated lid using the following cycling conditions: 72 °C for 5 min; 98 °C for 30 s; 14 cycles of 98 °C for 10 s and 63 °C for 30 s; final extension at 72 °C for 1 min and hold at 8 °C. The library fragments were purified with XP beads (Beckman Coulter, Beverly, USA). The size distribution of libraries was determined by Agilent 4200 TapeStation analysis, and libraries were mixed to achieve equal representation as desired, aiming for a final concentration as recommended by the manufacturer. Sequencing was performed on the Illumina Novaseq 6000 using 150 bp paired-end following the manufacturer’s instructions.

Raw reads of the fastq format were first processed through in-house scripts. All the downstream analyses were based on high-quality clean data. The clean reads were then aligned to reference genome sequences using the BWA program. The bam file generated by the unique mapped reads as an input file, using the MACS2 software for callpeak with a cutoff q value < 0.05. Peaks were annotated using Homer’s annotatePeaks.pl. Count the results of the annotations and plot the distribution results using R. The Homer’s findMotifsGenome.pl tool was used for Motif analysis.

### RNA-seq and data analysis

RNA was harvested from 1 × 10^6^ cells in triplicate and stored in RNAlater RNA stabilization solution (ThermoFisher Scientific). RNA purification, quantification and qualification, library construction and transcriptome sequencing were performed at Jiayin Biotechnology Ltd. (Shanghai, China) according to the manufacturer’s instructions (Illumina, San Diego, CA). Briefly, RNA was isolated using Trizol reagent. mRNA was purified from total RNA using poly-T oligo-attached magnetic beads. Fragmentation was carried out using divalent cations under elevated temperature in NEBNext. First strand cDNA was synthesized using a random hexamer primer and M-MuLV Reverse Transcriptase (RNase H-). Second strand cDNA synthesis was subsequently performed using DNA Polymerase I and RNase H. Remaining overhangs were converted into blunt ends via exonuclease/polymerase activities. After adenylation of the 3’ ends of DNA fragments, NEBNext Adaptor with a hairpin loop structure was ligated to prepare for hybridization. In order to select cDNA fragments of preferentially 250 ~ 300 bp in length, the library fragments were purified with AMPure XP system (Beckman Coulter, Beverly, USA). Then 3 µl USER Enzyme (NEB, USA) was used with size-selected, adaptor-ligated cDNA at 37 °C for 15 min followed by 5 min at 95 °C before PCR. Then PCR was performed with Phusion High-Fidelity DNA Polymerase, Universal PCR primers and Index (X) Primer. Finally, PCR products were purified (AMPure XP system), and library quality was assessed on the Agilent Bioanalyzer 2100 system. The clustering of the index-coded samples was performed on a cBot Cluster Generation System using TruSeq PE Cluster Kit v3-cBot-HS (Illumia) according to the manufacturer’s instructions. After cluster generation, the library preparations were sequenced on an Illumina Novaseq6000 platform, and 150 bp paired-end reads were generated. After quality control, STAR was used to align clean reads to the reference genome. HTSeq v0.6.0 was used to count the read numbers mapped to each gene. Then the FPKM of each gene was calculated based on the length of the gene and reads count mapped to this gene. We applied the DESeq2 algorithm to filter the differentially expressed genes, after the significant analysis and FDR analysis under the following criteria: (i) |log2FC| > 1; (ii) Pvalue < 0.05.

### Patient-derived organoid culture

Patien-tderived organoids (PDOs) were performed as previously described [34, 35]. Upon arrival, ovarian cancer tissues were rinsed in cold PBS with penicillin/streptomycin (GIBCO, 15140-122) for five cycles of five minutes each. Subsequently, the tissues were finely minced into fragments in a sterile dish on ice. Then tissue fragments underwent enzymatic digestion in an 8 mL digestion medium containing 7 mL DMEM medium (GIBCO, C1199500BT), 500 U/mL collagenase IV (Sigma-aldrich, C9407), 1.5 mg/mL collagenase II (Solarbio, C8150), 20 µg/mL hyaluronidase (Solarbio, h8030), 0.1 mg/mL dispase type II (Sigma-aldrich, D4693), 10 µM RHOK inhibitor ly27632 (Sigma-aldrich, Y0503) and 1% fetal bovine serum on an orbital shaker at 37 °C for 30–60 min. Tumor cells were isolated by centrifugation at 300–500 g for 5 min and seeded into Matrigel in a well of pre-warmed 24-well flat bottom cell culture plate (Costar, 3524) and overlayed with 500 µL PDO culture medium after incubation in a 37 °C and 5% CO2 culture incubator for 5–8 min.

The PDO culture medium was refreshed every three days, and PDOs were monitored and photographed at appropriate intervals. Typically, organoids were passaged every 1–2 weeks. For passaging, organoids were gently pipetted out of Matrigel using cold PBS and then mechanically sheared through a 1% BSA-coated pipette tip. Following these steps, the organoids were washed several times with centrifugation at 200–300 g until Matrigel was cleared out. Organoid fragments were suspended in Matrigel and seeded as described above. Cryopreservative medium (serum free) (CELLBANKER™ 2, ZENOAQ, 170,905) was used for organoids cryopreservation. 10 µM RHOK inhibitor ly27632 must be supplemented to the culture medium for organoid resuscitation.

### PDO preparation for drug tests

Human ovarian cancer organoids were prepared as previously described. Organoids were harvested and seeded in a 96-well cell culture plate (Corning, 3799) when organoids grew to 50 μm in diameter. The sandwich method was used for drug tests. Before seeding, 50 µL 50% Matrigel (Corning, 356,231, Matrigel mixed with PBS 1:1) was dropped on the bottom of the culture plate as the bottom layer. Then, 10 µL 10% Matrigel (Matrigel mixed with PBS 1:9) containing 50 ± 20 organoids was dropped on the bottom layer as the middle layer. Organoid culture medium (200 µL) was added to each well as the upper layer. Drug tests would start after one day of culturing.

### Transcriptional activity

Cells grown to 60–80% confluency were trypsinized and seeded in 24-well culture plates. After 24 h, they were transfected with 500 ng of the indicated DOT1L promoter sequence cloned into the PGL4.0 reporter plasmid. Transfection was performed with Hieff Trans® Liposomal Transfection Reagent (Yeasen, Shanghai, China) following the manufacturer’s instructions. The activities of firefly and Renilla luciferases were measured as relative luminescence units (RLU) using the Dual Luciferase Reporter Assay System (Yeasen, Shanghai, China) 48 h after transfection. Firefly RLU values were normalized to Renilla RLU values and an empty reporter vector. Triplicate samples were systematically included, and experiments were repeated at least three times. The results are shown as mean values with their respective standard deviations.

### Tumor formation assay in nude mice

Female BALB/c nude mice aged 4–6 weeks (Shanghai SLAC Laboratory Animal Co., Ltd.; Shanghai, China) were raised in a pathogen-free environment with a 12-h day-night cycle (Department of Laboratory Animal Science in Shanghai Medical College of Fudan University). 100 µL ovarian cancer cells (4 × 10^6^ cells) were subcutaneously injected into the left armpit of each mouse. Olaparib was dissolved in DMSO and diluted to 5 mg/mL with PBS before injection, and 10% DMSO in PBS was used as the vehicle control. When the tumor volumes reached ~ 50mm^3^, the mice were evenly divided into four groups, and Olaparib (50 mg/kg) and SGC0946 (50 mg/kg) were orally administered to mice three times per week separately or together for 4–6 weeks. The tumor was measured at the indicated time points and was calculated by the formula π/6 × length × width^2^.

### ChIP assay, qPCR and RT-qPCR

A ChIP assay was performed using SimpleChIP® Enzymatic Chromatin IP Kit (Magnetic Beads) (9003 S, CST) according to the manufacturer’s instructions. SKOV-3 cells were cross-linked with 1% formaldehyde and then washed with cold PBS, lysed with the lysis buffer, and then sonicated to produce an average DNA length of 500-1,000 bp. Immunoprecipitation was then performed with the indicated antibodies. Purified DNA fragments were analyzed by qPCR using 2×SYBR Green Pro Taq HS Premix (AG11702, Accurate Biology) on a LightCycler 480 Real-Time system (Roche), and precipitated DNA was calculated as a percentage of input DNA. RNA extraction was performed using TRIzol reagent (Life Technologies, Waltham, MA, USA), and cDNA was prepared using Evo M-MLV RT Master Mix Kit (AG11706, Accurate Biology, China). The primers used for the ChIP assay and qPCR are listed in Supplementary Table S1.

### Immunohistochemistry (IHC) staining

Totally 273 Chinese patients diagnosed with high-grade serous ovarian carcinoma were involved in this study. All patients had surgical resections at Fudan University Shanghai Cancer Center (FUSCC). Informed consent was obtained from all patients, and the use of clinical samples in this study was approved by the ethics committee of FUSCC. The tumor tissues were fixed with formalin and embedded in paraffin. Following deparaffinization in xylene, rehydration in graded ethanol, and heat-induced antigen retrieval, 4–6-µm-thick tissue sections were incubated with primary antibodies (1:800) at 4 °C overnight, followed by incubation with the corresponding secondary antibodies, visualization using DAB (ZSGB-BIO, Beijing, China), and counterstaining with hematoxylin. The scoring system was based on the staining intensity and extent, as follows: 0 (negative), 1 (weakly positive), 2 (moderately positive), and 3 (strongly positive). The staining positive rate score was calculated as: 1 (0–25%), 2 (26–50%), 3 (51–75%), and 4 (76–100%). The IHC grade was calculated as follows: staining intensity score × positive proportion score. The final score for each sample was the average score for two duplicates. Survival curves were calculated according to the Kaplan-Meier method; survival analysis was performed using the log-rank test.

### Quantification and statistical analysis

Statistical analysis was performed using GraphPad Prism v8.0. Statistical significance was determined by the unpaired, two-tailed Student’s t-test. Survival analyses were determined by the Kaplan-Meier curve and log-rank test. All data represent the mean ± SD. P-values were demonstrated in the graphs using * for *P* < 0.05, ** for *P* < 0.01, *** for *P* < 0.001, and **** for *P* < 0.0001. ns. represents not significant.

## Results

### Upregulated DOT1L expression correlates with PARPi resistance in OC

**Fig. 1:**
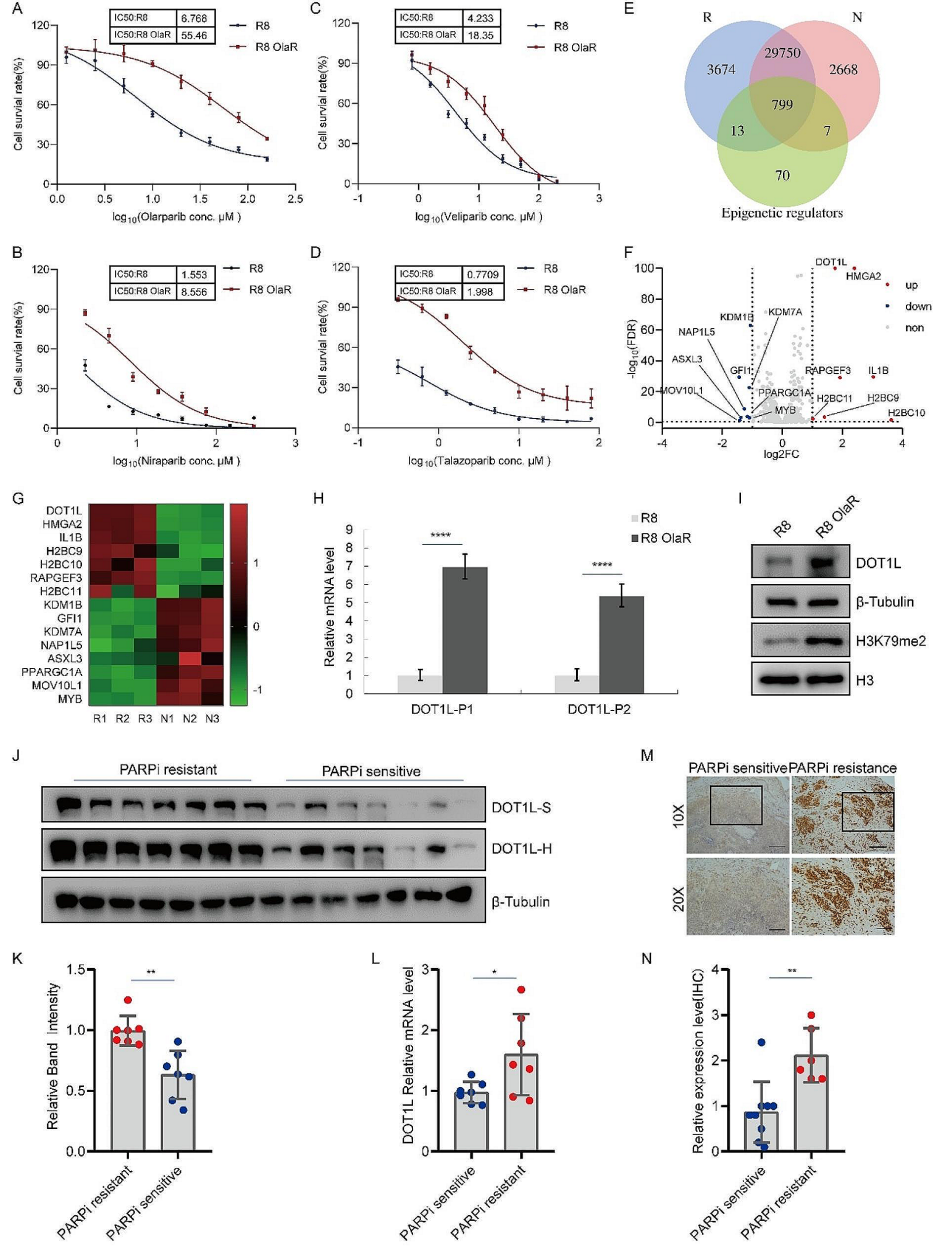
Upregulated DOT1L expression correlates with PARPi resistance in OCA. The non-BRCA mutated cell lines OVCAR8 were subjected to a gradual increase in the concentration of Olaparib (from 0.5 to 20 µM) to allow for the development of acquired resistance. The IC50 values of Olaparib-resistant OVCAR8 (R8 OlaR) and original parent OVCAR8 (R8) cell lines were detected by CCK8 assay. **B**. Niraparib IC50 curves of parent OVCAR8 and cells with acquired resistance to Olaparib. **C**. Veliparib IC50 curves of parent OVCAR8 and cells with acquired resistance to Olaparib. **D**. Talazoparib IC50 curves of parent OVCAR8 and cells with acquired resistance to Olaparib. **E**. RNA-seq was performed on R8 OlaR (R) (*n* = 3) and R8 (N) (*n* = 3). Venn diagram illustrating the overlap between the RNA-seq data (R and N) and the classic epigenetic regulator genes. **F**. Volcano plot showing differential expression of mRNAs among overlap gene in E. Red dots represent differently expressed mRNAs with *P* < 0.05 and Log2FC > 1; blue dots represent mRNAs with *P* < 0.05 and Log2FC<-1; grey dots represent mRNAs with no significance. **G**. Heatmap showing differentially expressed epigenetic-related genes between R and N (F). H. DOT1L mRNA levels in R8 OlaR and its original parent OVCAR8 cells were analyzed by RT-qPCR. **I**. R8 OlaR and its original parent OVCAR8 cells were collected and subjected to western blotting to detect with the indicated antibodies. **J**-**K**. Analysis of DOT1L protein levels in PARP inhibitor-resistant (*n* = 7) and sensitive fresh-frozen (FF) tissue tissues (*n* = 7) (**J**). Quantified results are presented as the means ± SD (*n* = 3), **p < 0.01 (**K**). **L**. DOT1L mRNA levels in PARPi-resistant and sensitive OC tissues were analyzed by RT-qPCR. The data is presented as the means ± SD, *p < 0.05. **M** IHC staining of DOT1L in PARPi-resistant and sensitive OC tissues. Representative images are shown. Scale bars: 200 μm (upper); 100 μm (lower) (left). Quantification of DOT1L expression in PARPi-resistant OC tissues (*n* = 6) and sensitive tissues (*n* = 9), **p < 0.01 (right)

In order to characterize the specific epigenetic regulators that contribute to PARPi resistance, the Olaparib resistance cells OVCAR8 (R8 OlaR) were constructed, as shown in Fig. 1A. The half-maximal inhibitory concentration (IC50) value for Olaparib treatment in R8 Ola-R cells was determined to be 55.46 µM, in comparison to 6.768 µM in the original parent OVCAR8 (R8) cell line. Three additional PARP inhibitors, Niraparib, Veliparib, and Talazoparib, were also evaluated in R8 OlaR and R8 cells, where a similar increase in IC50 was observed as with Olaparib (Fig. 1B-D). This indicated that R8 OlaR cells exhibited widespread PARPi resistance. Subsequently, RNA-seq analysis was performed to analyze the cells’ gene regulation profiles. As shown in Fig. S1A and B, the Pearson correlation and principal component analysis (PCA) among the R8 OlaR (R) and R8 (N) groups revealed significant correlations among the similar groups and heterogeneity between R and N. To identify potential epigenetic regulators associated with PARPi resistance, we integrated RNA-seq data with the classic epigenetic regulator gene pool and identified 799 intersecting genes (Fig. 1E). Further analysis of gene expression differences indicated that DOT1L and HMGA2 were upregulated most significantly (Fig. 1F and G). HMGA2 has been previously reported to act as a functional antagonist of PARP1 inhibitors in tumor cells [36], while DOT1L, a predictor of poor prognosis in most solid tumors, remains relatively understudied, with its role in PARP inhibitor-resistant OC still unidentified. Consequently, we selected DOT1L as the most promising candidate target for further investigation.

To verify the sequencing results, we performed RT-qPCR in R8 OlaR and R8 cells. We observed increased DOT1L mRNA expression levels in R8 OlaR cells compared to R8 cells (Fig. 1H). In addition, the protein expression of DOT1L in R8 OlaR cells exhibited a significantly higher level than that of the original parent OVCAR8 cells (Fig. 1I). Furthermore, DOT1L was further identified in Olaparib-resistant SKOV-3 (SV3 OlaR) cells, which exhibited cross-resistance to other PARPis too (Fig. S1C-H). We also measured DOT1L mRNA and protein expression levels and found that the mRNA and protein expression levels increased in PARPi-treated cells (Fig. S1I-L). Finally, the OC cells and their cisplatin-resistant (DDP) counterparts were constructed (Fig. S1M), and the protein and mRNA levels of DOT1L were measured in these cells, as shown in Fig. S1N-O. The results demonstrated that there was no significant increase in DOT1L mRNA and protein expression levels in cisplatin-resistant OC cells. This suggests that the upregulation of DOT1L is associated with PARPi-acquired resistance in OC. Particularly, the upregulation of DOT1L is induced by PARPi and is dependent on PARP1.

Given that DOT1L was expressed at high levels in PARPi-resistant cells and correlated with PARPi resistance, we decided to verify our results further through clinical samples. We measured the DOT1L mRNA and protein levels in the PARPi-resistant and sensitive fresh-frozen (FF) tissue samples. The results showed that DOT1L protein expression levels and mRNA levels were significantly elevated in PARPi-resistant samples in comparison to those in PARPi-sensitive samples (Fig. 1J-L), which is consistent with the cellular results. Furthermore, the DOT1L expression level was evaluated in PARPi-resistant and sensitive OC tissues by immunohistochemistry (IHC) staining. The results demonstrated that DOT1L was highly expressed in PARPi-resistant tissues as well (Fig. 1M-N). This provides additional evidence supporting our hypothesis that highly expressed DOT1L is correlated with PARP inhibitor resistance in OC. Finally, we examined the levels of DOT1L by IHC staining in a human tissue array, which included 283 OC samples from patients. We observed a reduction in overall and progress-free survival in patients with highly expressed DOT1L levels (Fig. S2A-C). Furthermore, the highly expression of PARP1 postulated to be associated with poor overall or progress-free survival of OC patients (Fig. S2D-F), consistent with the results analyzed in the TCGA data (Fig. S2G-H). The correlation of DOT1L expression and clinicopathological parameters in ovarian cancer tissues also revealed a significant correlation between DOT1L and poor prognosis in OC (Fig. S2I). This indicated that DOT1L expression was significantly associated with chemotherapeutic response and was an independent prognosticator of progression-free and overall survival in OC patients.

### DOT1L regulates OC sensitivity to Olaparib and contributes to PARPi resistance

**Fig. 2:**
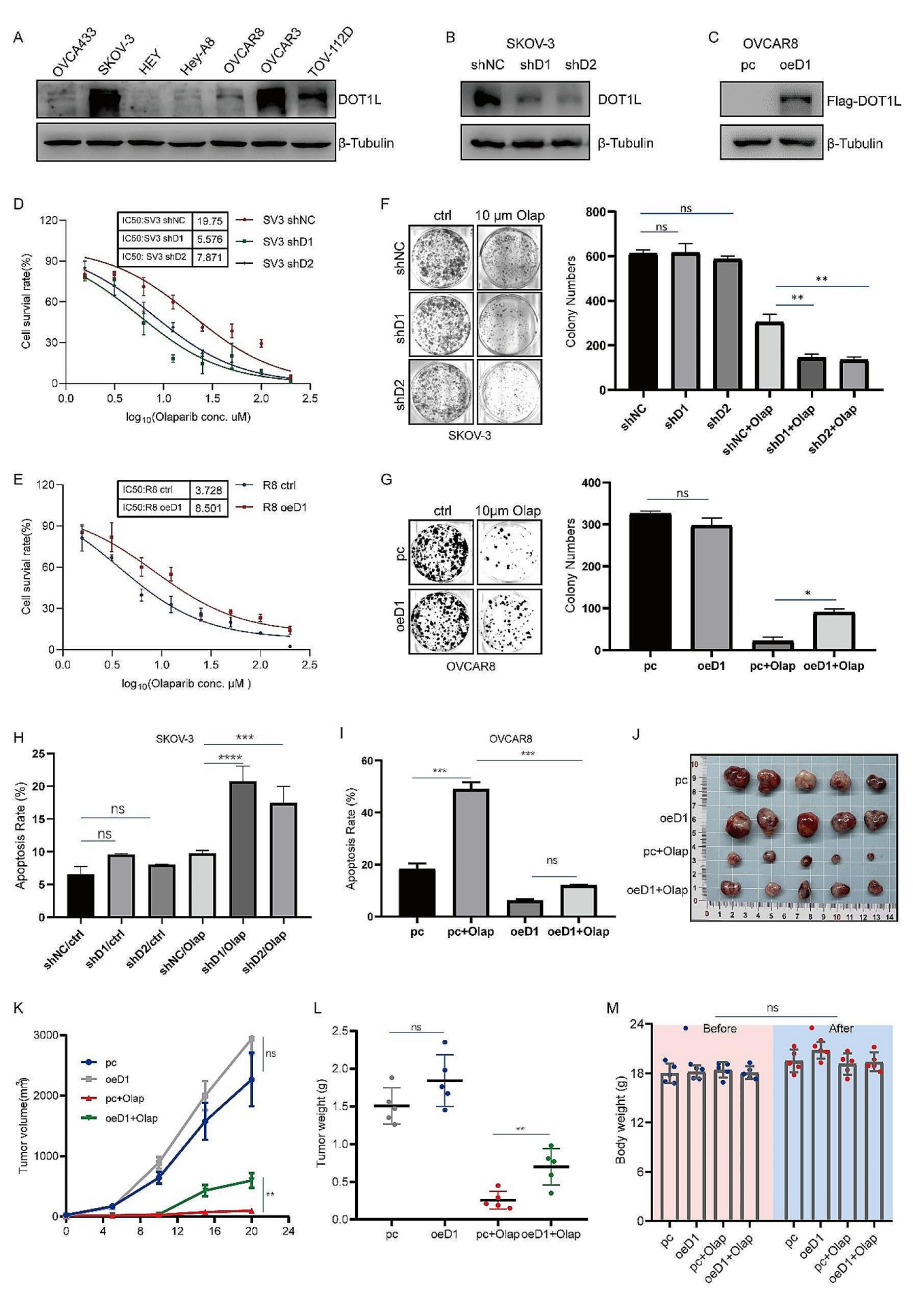
DOT1L regulates OC sensitivity to Olaparib and contributes to PARPi resistance. **A**. DOT1L protein levels in a panel of OC cell lines were examined by western blotting. **B**. PLKO.1 and DOT1L shRNA (shDOT1L) plasmids were stably transfected into SKOV-3 cell line. Western blotting was used to determine DOT1L protein levels. **C**. PCMV, and PCMV DOT1L plasmids were stably transfected into OVCAR8 cell line. Western blotting was used to determine DOT1L protein levels. **D**. The CCK8 assay was performed to detect cell viability in SKOV-3 cells treated with Olaparib (Olap) for 96 h. **E**. The CCK8 assay was performed to detect cell viability in OVCAR8 cells treated with Olaparib for 96 h. **F**. Clonogenic assays were conducted to assess the colony formation efficiency of SKOV-3 cells in the presence of Olaparib for 7–14 days (left). The number of clones was quantified (right). **G**. Clonogenic assays were conducted to assess the colony formation efficiency of OVCAR8 cells in the presence of Olaparib for 7–14 days (left). The number of clones was quantified (right). **H**. A flow cytometry assay was performed to detect cell apoptosis in SKOV-3 cells treated with Olaparib (10 µM) for 48 h. **I**. A flow cytometry assay was performed to detect cell apoptosis in OVCAR8 cells treated with Olaparib (10 µM) for 48 h. (Data is presented as the mean ± SD; ns, *p* > 0.05; *p < 0.05, **p < 0.01, ***p < 0.001; ****p < 0.0001, *n* = 3). J-L. OVCAR8 and DOT1L stably overexpressed OVCAR8 cells (4 × 10^6^ cells) were subcutaneously injected into the left armpit of each mouse. When the tumor volumes reached approximately 50 mm^3^, the mice were randomly divided into four groups (pc + PBS, pc + Olap, oeDOT1L + PBS, oeDOT1L + Olap) and received an intraperitoneal injection of Olaparib (Olap, 50 mg/kg) or PBS three times a week. Three weeks post-injection, the mice were sacrificed, and their body weights and tumor weight were quantified. Tumors from each group are shown in (**J**). Tumor growth curve (K) and tumor weights of each group (**L**) were quantified. **M**. The nude mice’s body weights of each group before and after administration. (Data are presented as the mean ± SD, ns, *p* > 0.05; *p < 0.05; **p < 0.01, *n* = 5)

In order to demonstrate the functional significance of DOT1L in PARPi resistance in OC, we next assessed whether DOT1L regulates OC sensitivity to Olaparib and contributes to PARPi resistance. Firstly, we detected DOT1L protein levels in different cell lines originating from OC tissues (Fig. 2A). DOT1L stable knockdown cells were constructed in DOT1L high expressed cells SKOV-3 and OVCAR3 (Fig. 2B, Fig. S3A). DOT1L stable overexpressed cells were conducted in DOT1L low expressed cells OVCAR8 and OVCA433 (Fig. 2C, Fig. S3B), Then we conducted an Olaparib IC50 assay and colony formation assay in DOT1L knockdown SKOV-3 or OVCAR3 cell line and DOT1L overexpressed OVCAR8 or OVCA433 cell line. Results showed that the knockdown of DOT1L significantly enhanced the sensitivity of SKOV-3 and OVCAR3 cells to Olaparib (Fig. 2D, Fig. S3C). By contrast, cells with DOT1L overexpression showed increased cell viability following Olaparib treatment (Fig. 2E, Fig. S3D). Correspondingly, cells with DOT1L knockdown exhibited a dramatically decreased clonogenic ability in response to Olaparib treatment, but not in the control condition alone (Fig. 2F, Fig. S3E). In contrast, cells with DOT1L overexpression exhibited the opposite result (Fig. 2G, Fig. S3F).

In addition, we conducted a flow cytometry assay in these cells to further examine the effect of DOT1L on PARPi sensitivity. As illustrated in Fig. 2H, Fig. S3G, knockdown of DOT1L protein was found to significantly enhance Olaparib-induced apoptosis. On the contrary, a decreased proportion of apoptotic cells following Olaparib treatment was observed in DOT1L overexpression cells (Fig. 2I, Fig. S3H). Interestingly, the apoptosis did not exhibit a difference upon DOT1L interference alone. Next, we assessed whether aberrant DOT1L expression interfered with the tumor response to Olaparib treatment in a xenograft mouse model. BALB/c nude mice were subcutaneously injected with OVCAR8 cells transfected with PCMV (pc) or PCMV DOT1L (oeD1). Approximately one week later, the mice were equally divided into four groups and intraperitoneally injected with 50 mg/kg of Olaparib for three weeks. The tumor volumes and tumor weight in the oeD1 group were significantly higher than those in the pc group (Fig. 2J-L), and the mouse body weights of each group remained unchanged before and after administration (Fig. 2M). These findings suggest that DOT1L may regulate OC sensitivity to Olaparib and confer PARPi resistance in vitro and in vivo.

### PARP1-mediated transcription regulation directly influences DOT1L expression

**Fig. 3:**
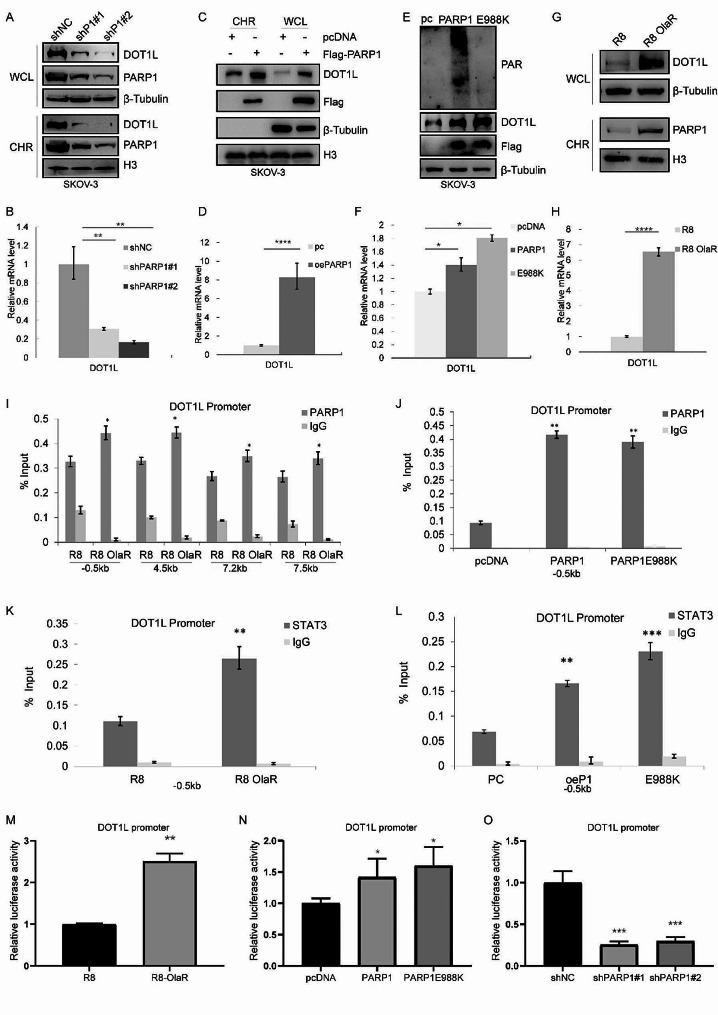
PARP1-mediated transcription regulation directly influences DOT1L expression. **A** PLKO.1, PARP1 shRNA (shPARP1) plasmids were stably transfected into SKOV-3 cells. Whole cell lysate (WCL) and chromatin-binding protein (CHR) were extracted and analyzed by western blotting with the indicated antibodies. **B**. RT-qPCR was used to determine the DOT1L and PARP1 mRNA levels. **C**. PCMV, PCMV PARP1 plasmids were stably transfected into SKOV-3 cells. Whole cell lysate (WCL) and chromatin bind protein (CHR) were extracted and analyzed by western blotting with the indicated antibodies. **D**. Quantification of PARP1 and DOT1L mRNA levels in (**C**). **E**. SKOV-3 cells were transfected with control pcDNA, Flag-PARP1(WT), or enzymatically defective Flag-PARP1 (PARP1 E988K). Western blotting was performed to detect DOT1L protein expression levels. **F**. DOT1L mRNA levels in pcDNA-, PARP1(WT)-, or PARP1 E988K-transfected SKOV-3 cells were analyzed by RT-qPCR. The data represents the means ± SD (*n* = 3). *p < 0.05. **G**-**H**. OVCAR8 and OVCAR8 OlaR cells were collected, and western blotting and RT-qPCR were performed to detect DOT1L protein expression(G) and mRNA (**H**) levels. **I**. ChIP–qPCR showing the level of the indicated proteins recruited to the DOT1L promoter regions. The data represents the means ± SD (*n* = 3). *p < 0.05. Four independent sets of DOT1L primers were used. **J**. PARP1-ChIP assay was performed in pcDNA-, PARP1(WT)-, or PARP1 E988K- transfected SKOV-3 cells to examine PARP1 occupancy at DOT1L. **K**. STAT3-ChIP assay was performed in OVCAR8 and OVCAR8 OlaR cells to examine STAT3 occupancy at DOT1L. L. STAT3-ChIP assay was performed in pcDNA-, PARP1(WT)-, or PARP1 E988K- transfected SKOV-3 cells to examine STAT3 occupancy at DOT1L. **M**. OVCAR8 and OVCAR8 OlaR cells were transfected with the DOT1L promoter report gene. The luciferase activity was measured 36 h after transfection. **N**. SKOV-3 cells were transfected with the DOT1L promoter report gene, together with control pcDNA, wild-type Flag-PARP1, and mutant Flag-PARP1 E988K as indicated. The luciferase activity was measured 36 h after transfection. **O**. The luciferase reporter assays were performed in PARP1 stably knockdown SKOV-3 cells

To probe the mechanism of PARPi-induced high DOT1L expression, we sought to ascertain whether PARP1 interference could phenocopy PARPi-induced DOT1L expression. Chromatin-bound proteins and whole cell lysate were extracted and analyzed using western blot. We found that stable knockdown of PARP1 in SKOV-3 and OVCAR3 cells resulted in a general decrease of DOT1L protein and mRNA expression (Fig. 3A-B, Fig. S4A-B). Conversely, a significant elevation of DOT1L protein and mRNA expression was observed in PARP1-overexpressed SKOV-3 and OVCAR3 cells (Fig. 3C-D, Fig. S4C-D). The TCGA data analysis also showed a positive correlation between DOT1L and PARP1 (Fig. S4E). Considering that PARP1 controls the transcription of target genes, either with both catalytic-dependent and catalytic-independent mechanisms, we established a PARP1 catalytic activity deletion plasmid (PARP1 E988K) and transfected it into SKOV-3 cells. As shown in Fig. 3E-F, increased expression of DOT1L in both PARP1 WT and PARP1 E988K-transfected cells was observed. Furthermore, we verified the chromatin binding of PARP1 in R8 OlaR cells and the total PARP1 protein and mRNA expression level. As shown in Fig. 3G-H, Fig. S4F-G, the increased PARP1 binding to chromatin and DOT1L expression level in R8 OlaR were observed, yet the expression of PARP1 protein and mRNA did not exhibit a significant change, indicating that PAPR1 DNA trapping induced by PARPi treatment resulted in increased PARP1 levels among chromatin-bound proteins and induced increased DOT1L expression. Besides, we detected the half-life of DOT1L in PARP1 knockdown, PARP1 overexpressed, and R8 OlaR cells. Our findings demonstrated that PARP1 had no effect on DOT1L half-life period or protein stability (Fig. S4H). Moreover, we examined whether DOT1L could affect PARP1 expression in a reciprocal manner. As shown in Fig. S4I-K, PARP1 expression levels exhibited no significant change in DOT1L-interfered cells. These results indicated that PARP1 could regulate DOT1L expression through transcription regulation in a catalytic-independent manner.

The role of PARP1 as a transcription factor has been well documented, and PARP1 nucleosome occupancy regulates transcriptional outcomes through various mechanisms [37, 38]. To this end, we ask whether PARP1 regulates DOT1L expression directly. We examined the direct binding of PARP1 on DOT1L promoter by ChIP-qPCR. In R8 OlaR cells, we conducted ChIP by pulling down with PARP1 antibody, followed by qPCR using four sets of primers surrounding DOT1L promoter. These primers span 0.5 kb upstream of the transcription start site (TSS) and 7.5 kb downstream of the TSS. As shown in Fig. 3I, we detected enrichment of PARP1 on DOT1L promoter at R8 OlaR cells compared to the original parent OVCAR8 cell line by all four sets of primers. This indicated that PARPi treatment induced PARP1 DNA trapping, resulting in increased PARP1 binding to the DOT1L promoter and subsequent promotion of DOT1L transcription. To reinforce this hypothesis, we conducted ChIP-qPCR in PARP1 WT and PARP1 E988K-transfected SKOV-3 cells by pulling down PARP1, directly binding with primers set-1 (-0.5 kb) and set-2 (-4.5 kb). In PARP1 WT- and PARP1 E988K-transfected cells, enrichment of PARP1 on DOT1L promoter was increased similarly compared to the control (Fig. 3J, Fig. S4L). These findings suggest that PARP1 directly binds to the DOT1L promoter and regulates DOT1L transcription.

Considering STAT3 as a transcription factor that participates in DOT1L transcription [39], we performed ChIP-qPCR in R8 and R8 OlaR cells to detect the enrichment of STAT3 on DOT1L promoters. As shown in Fig. 3K, the binding of STAT3 on the DOT1L promoter was higher in R8 OlaR cells. Moreover, in PARP1 WT- and PARP1 E988K-transfected cells, the enrichment of STAT3 on the DOT1L promoter was similarly increased compared to the control (Fig. 3L). To further test if PARP1 enhanced the transcription of DOT1L, we cloned the promoter region of DOT1L downstream of a luciferase reporter gene (pGL4.14-DOT1L). The luciferase activity of the cells transfected with pGL4.14-DOT1L was significantly increased in R8 OlaR cells (Fig. 3M). A similar phenomenon was observed in PARP1WT and PARP1 E988K-transfected cells (Fig. 3N, Fig. S4M). On the contrary, the luciferase activity was decreased in PARP1 knockdown cells (Fig. 3O, Fig. S4N). These data suggest that PARP1 participates in DOT1L transcription regulation independent of its catalytic activity, and PARPi has been shown to increase PARP1 and STAT3 binding to the DOT1L promoter, which in turn induces DOT1L upregulation.

### DOT1L facilitates PARPi resistance via H3K79 methylation

**Fig. 4:**
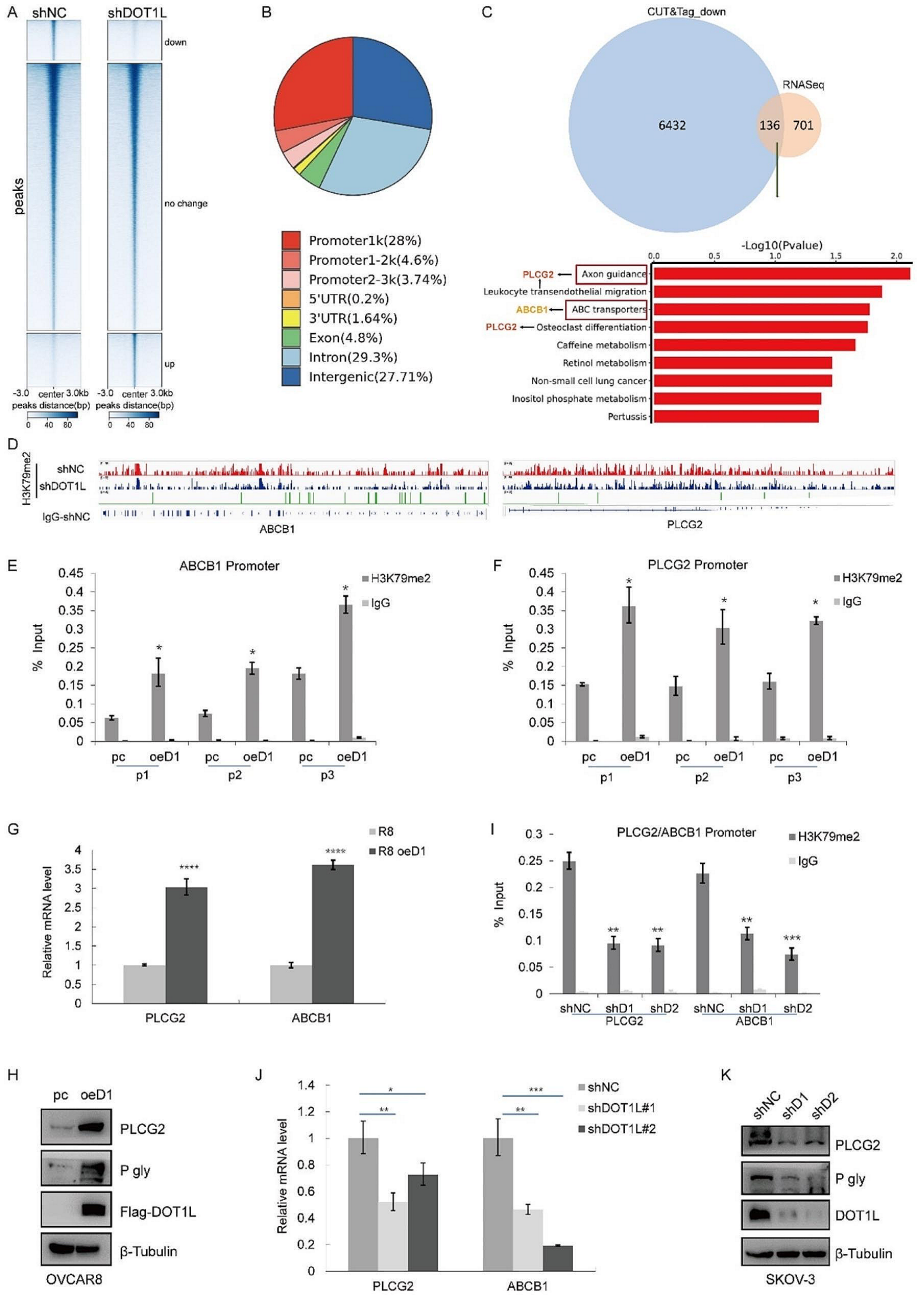
DOT1L facilitates PARPi resistance via H3K79 methylation. **A**. Heatmaps of H3K79me2 levels detected by CUT&Tag around gene body regions in control (shNC) and DOT1L knockdown (shDOT1L) SKOV-3 cells treated with Olaparib 10µM for 48 h. 3 kb windows spanning the TSS to TES of all genes were plotted. Genes were arranged by their enrichment of H3K79me2 in shNC and shDOT1L cells. **B**. The distributions of H3K79me2-binding regions are shown in the pie charts. **C**. Venn diagram showing the overlap between RNA-seq data and CUT&Tag data. The KEEP analysis revealed the significantly enriched items based on H3K79me2 signature. **D**. IGV tracks showing the enrichment of H3K79me2 in ABCB1 and PLCG2 gene regions in control (shNC) and DOT1L knockdown (shDOT1L) SKOV-3 cells treated with Olaparib 10µM for 48 h. **E**-**F**. ChIP–qPCR showing the level of the indicated proteins recruited to the PLCG2 (**E**) and ABCB1 (**F**) promoter regions in DOT1L-overexpressed OVCAR8 cells. The data represent the means ± SD (*n* = 3). *p < 0.05. three independent sets of PLCG2 and ABCB1 primers were used. **G**. RT-qPCR was performed in DOT1L overexpressed OVCAR8 cells to determine PLCG2 and ABCB1 mRNA levels. H. PLCG2 and ABCB1 (P-gly) expression was measured by western blotting in DOT1L overexpressed OVCAR8 cells. **I**. An H3K79me2-ChIP assay was performed in DOT1L knockdown SKOV-3 cells to examine H3K79me2 occupancy at PLCG2 and ABCB1. **J**. RT-qPCR was performed in DOT1L knockdown SKOV-3 cells to determine PLCG2 and ABCB1 mRNA levels. **K**. PLCG2 and ABCB1 (P-gly) expression was measured by western blotting in DOT1L knockdown SKOV-3 cells

DOT1L, as an H3K79me1/2/3 methylase, mediated H3K79me2 methylation, which has been identified as a transcription active maker in gene expression regulation [40, 41]. Here, to systematically investigate mechanisms of DOT1L-mediated PARPi resistance from transcription regulation levels, we conducted H3K79me2 CUT&Tag to profile the distributions of H3K79me2 in wild-type control (shNC) and DOT1L knockdown (shDOT1L) cells following Olaparib treatment to define the gene regulatory profiles in OC. CUT&Tag with IgG was performed in shNC and shDOT1L cells as the negative control for enrichment of H3K79me2. We analyzed the enrichment of H3K79me2 in the region encompassing 3 kb upstream and downstream of gene body regions, and the occupancy of H3K79me2 genome-wide across the shNC and shDOT1L cell lines was profiled (Fig. 4A, S5A). The genomic distribution of regions showing the H3K79me2 CUT&Tag signal further confirmed H3K79me2 as a transcription marker (Fig. 4B). To identify putative targets of DOT1L/H3K79me2 in OC, we integrated CUT&Tag data with RNA-seq data to identify 136 intersecting genes. KEGG analysis indicated that axon guidance was the most enriched crosstalk function, followed by leukocyte transendothelial migration, and ABC transporters (Fig. 4C, Fig. S5B). We selected the top genes among these pathways and measured their expression levels. As shown in Fig. S5C, the mRNA expression levels of PLCG2 and ABCB1 were significantly decreased in DOT1L knockdown SKOV-3 cells.

ABCB1, the key member of ATP-binding cassette (ABC) transporters, is related to exporting bound toxins out of the cell and could contribute to multidrug resistance in cancers subsequently [15]. PLCG2 (Phospholipase C, gamma 2) occurs in these pathways frequently (Fig. S5B), which are correlated to chemotherapy resistance in multiple cancers [16], including OC. Furthermore, the IGV (Integrative Genomics Viewer) revealed that H3K79me2 was enriched at the PLCG2 and ABCB1 promoters in the CUT&Tag (Fig. 4D). To corroborate these findings, an H3K79me2 ChIP assay was conducted, demonstrating that H3K79me2 was enriched on ABCB1 and PLCG2 promoters in DOT1L-overexpressed cells compared to original OVCAR8 by all 3 sets of primers (p1, p2, p3) (Fig. 4E-F). Consistent with these results, the mRNA and protein levels of ABCB1 and PLCG2 were also increased in DOT1L-overexpressed cells (Fig. 4G-H). By contrast, decreased binding of H3K79me2 to ABCB1 and PLCG2 promoters was observed in SKOV-3 DOT1L knockout cells (Fig. 4I), and the mRNA and protein levels of ABCB1 and PLCG2 were decreased in DOT1L knockdown cells accordingly (Fig. 4J-K). GEPIA also showed a positive correlation between DOT1L and PLCG2 or ABCB1 (Fig. S5D-E). All these results further strengthen the notion that ABCB1 and PLCG2 are downstream genes of DOT1L, which were considered to contribute to DOT1L-mediated PARPi resistance.

### PARP1-DOT1L-PLCG2/ABCB1 axis contributes to PARPi resistance

**Fig. 5:**
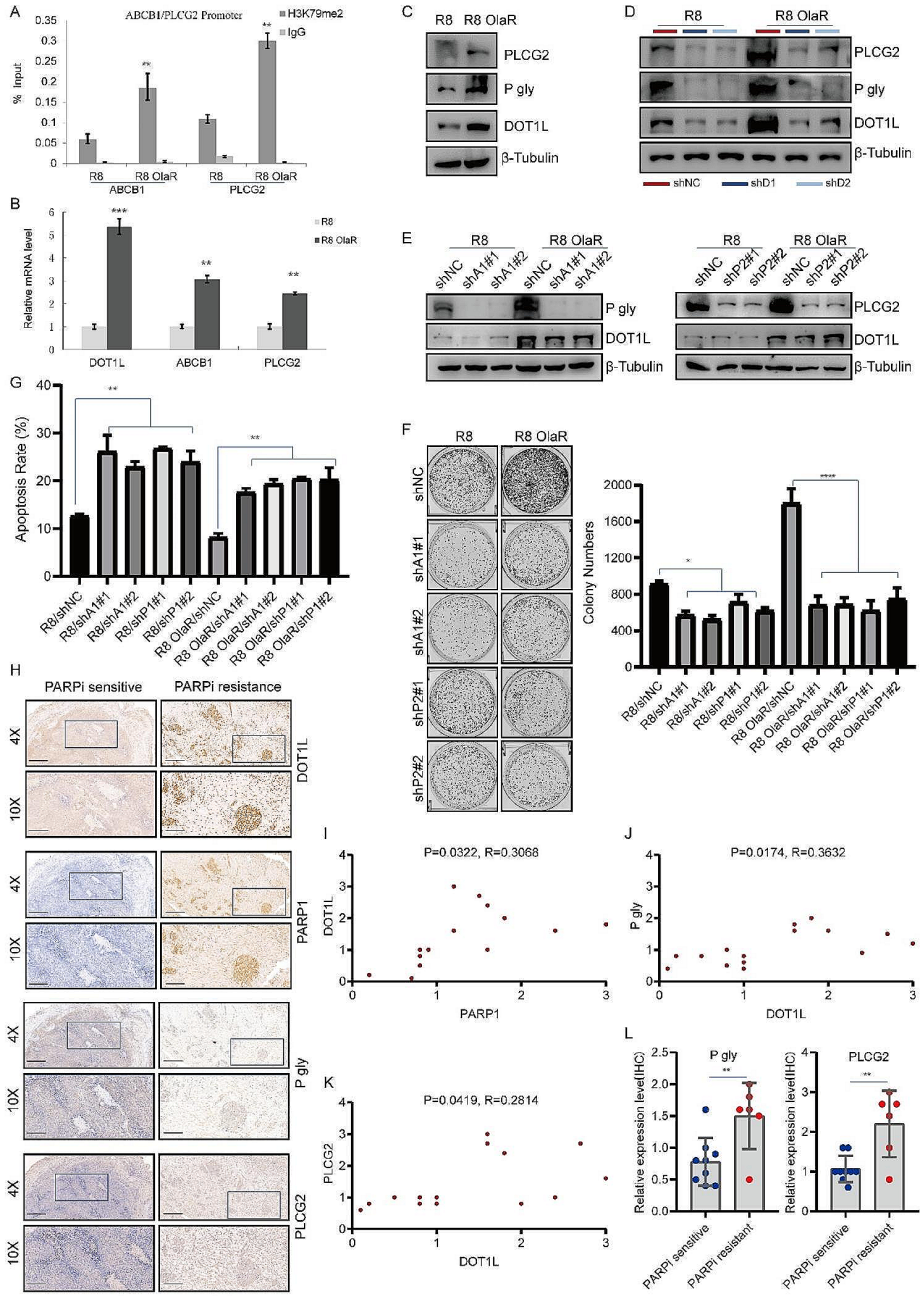
PARP1-DOT1L-PLCG2/ABCB1 axis contributes to PARPi resistance. **A**. H3K79me2-ChIP assay was performed with OVCAR8 Olaparib-resistant and parent OVCAR8 cell lines to determine H3K79me2 occupancy at PLCG2 and ABCB1. **B**. PLCG2 and ABCB1 mRNA levels were determined in R8 OlaR and parent OVCAR8 cells by RT-qPCR. **C**. Western blotting was performed in Olaparib-resistant OVCAR8 and parent OVCAR8 cell lines to examine PLCG2 and ABCB1 (P-gly) protein expression levels. **D**. Western blotting was performed in R8 OlaR and parent OVCAR8 cells which were transfected with shNC and DOT1L shRNA respectively with the indicated antibodies. **E**. R8 OlaR and parent OVCAR8 cells were transfected with shNC, ABCB1 shRNA and PLCG2 shRNA. After 72 h of transfection, cells were collected and analyzed by western blotting with the indicated antibodies. **F**–**G**. Colony formation (**F**) and cell apoptosis assay (**G**) were performed in R8 OlaR and parent OVCAR8 stably transfected cell lines. **H**. Immunohistochemistry (IHC) staining of DOT1L, PARP1, ABCB1 (P-gly), and PLCG2 in PARP inhibitor-resistant human ovarian carcinomas (OC) tissues and sensitive tissues. Representative images are shown. Scale bars: 400 μm (upper); 160 μm (lower). **I**–**K**. Correlation analysis between PARP1 and DOT1L(I), DOT1L and P-gly (**J**), and DOT1L and PLCG2 (**K**) were analyzed. **L**. Quantification of P-gly (right) and PLCG2 (left) expression in PARP inhibitor-resistant OC tissues (*n* = 6) and sensitive tissues (*n* = 9), **p < 0.01

As we identified that PLCG2 and ABCB1 are downstream of DOT1L/H3K79me2 in OC (Fig. 4), we next demonstrate the functional significance of the PARP1-DOT1L-PLCG2/ABCB1 axis in PARPi-resistant cells. Chip-qPCR was performed on R8 OlaR cells, and we found that H3K79me2 enrichment in PLCG2 and ABCB1 promoter regions was increased in R8 OlaR cells compared to parent OVCAR8 cells (Fig. 5A). Consistent with these results, the mRNA and protein levels of PLCG2 and ABCB1 were also increased in R8 OlaR cells (Fig. 5B-C). In addition, we performed ChIP-qPCR in SKOV-3 cells transfected with pcDNA, PARP1(WT), or PARP1(E988K) too. Here, we found that H3K79me2 enrichment in PLCG2 and ABCB1 promoter regions was increased in PARP1 WT- and PARP1 E988K-transfected cells compared to pcDNA-transfected cells (Fig. S6A-B). The mRNA and protein levels of PLCG2 and ABCB1 were also increased in PARP1 WT- and PARP1 E988K-transfected cells compared to pcDNA-transfected cells (Fig. S5C-D). To determine whether DOT1L-mediated PLCG2 and ABCB1 upregulation is indispensable in R8 OlaR and PARP1-overexpressed cells. We knockdown DOT1L in these cells and detect the protein expression levels of PLCG2 and ABCB1. As shown in Fig. 5D and Fig. S6E, DOT1L knockdown result in decreased expression of PLCG2 and ABCB1 in R8 OlaR and PARP1 overexpressed cells too.

Then, we knockdown the PLCG2 and ABCB1 genes in R8 OlaR cells (Fig. 5E). R8 OlaR shPLCG2 and shABCB1 cells were treated with Olaparib (10µM, 48 h) and collected to perform a colone formation assay and flow cytometry assay. As shown in Fig. 5F, PLCG2 and ABCB1 knockdown decreased the colone formation abilities of R8 OlaR cells in response to Olaparib. An enhanced apoptosis rate was also observed in these cells (Fig. 5G). In addition, we knocked out PLCG2 and ABCB1 in SKOV-3 cells (Fig. S6F). The flow cytometry assay performed in these cells showed that Olaparib-induced apoptosis was significantly increased in PLCG2 and ABCB1-knocked down SKOV-3 cells compared to control SKOV-3 cells (Fig. S6G). These results suggest that PLCG2 and ABCB1 contribute to OC cells being resistant to PARPi, and targeted inhibition of PLCG2 and ABCB1 could re-sensitize tumor cells that have acquired resistance to PARPi.

To further verify that DOT1L-induced accumulation of PLCG2 and ABCB1 contributes to PARPi resistance, we next knockout PLCG2 and ABCB1 genes in DOT1L-stably expressed OVCAR8 and parent OVCAR8 cells (Fig. S6H). These cells were treated with Olaparib, and subsequently applied to colony formation and flow cytometry assays. As shown in Fig. S6I-J, decreased colony-forming abilities and an enhanced apoptosis rate were observed in PLCG2 and ABCB1 knockdown cells, suggesting that PLCG2 and ABCB1 could abolish the DOT1L-induced PARPi resistance in OC cells. Finally, the expression of DOT1L, PARP1, PLCG2, and ABCB1 were analyzed in PARP inhibitor-resistant and sensitive OC tissues by IHC staining (Fig. 5H). We found a positive correlation between PARP1 and DOT1L, DOT1L and ABCB1, DOT1L and PLCG2 (Fig. 5I-K), and ABCB1 and PLCG2 were highly expressed in PARP inhibitor-resistant tissues as well (Fig. 5L). Therefore, our findings suggest that the PARP1-DOT1L-PLCG2/ABCB1 axis contributes to OC cells being resistant to PARPi.

### Targeted inhibition of DOT1L sensitizes OC to PARPi in vitro and in vivo

**Fig. 6:**
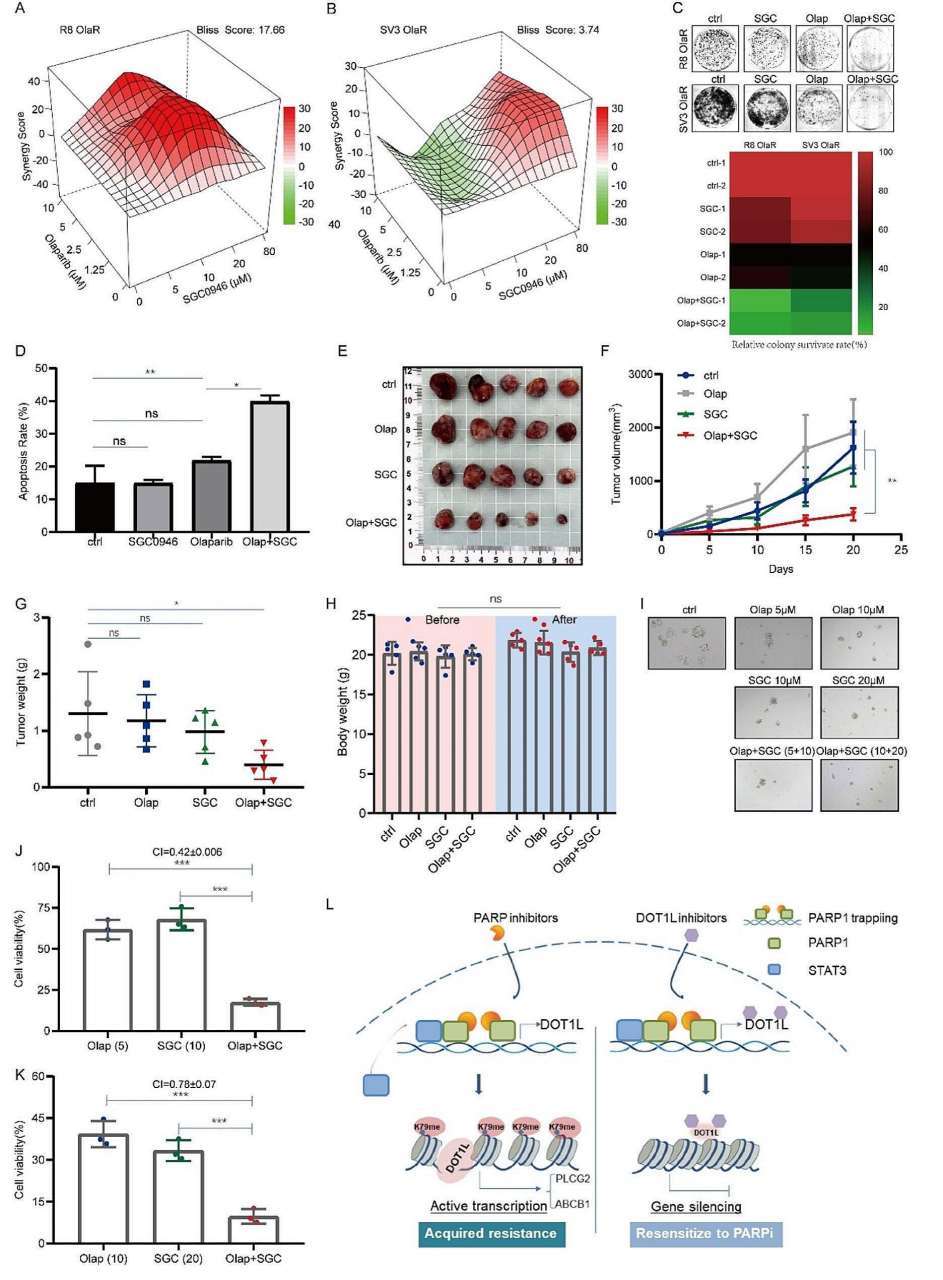
Targeted inhibition of DOT1L sensitizes OC to PARPi in vitro and in vivo. **A**–**B**. Bliss synergy analysis of SGC0946 and Olaparib treatment in R8 OlaR (**A**) and SV3 OlaR (**B**) cell lines. Synergy and antagonism degrees between the drugs were determined using SynergyFinder. A positive score represents a synergistic effect. **C**. A colony formation assay was conducted to detect the effect of combination therapy with SGC0946 (20µM) and Olaparib(10µM) on R8 OlaR and SV3 OlaR proliferation (upper). Quantification of the relative survival rate of clones (lower). **D**. A flow cytometry assay was performed to detect cell apoptosis in R8 OlaR cells treated with Olaparib (10 µM), SGC0946 (20µM), or Olaparib (10 µM) + SGC0946 (20µM) for 48 h. **E**. R8 OlaR cells (4 × 10^6^ cells) were subcutaneously injected into the left armpit of each mouse. When the tumor volumes reached approximately 50mm^3^, the mice were randomly divided into four groups (ctrl, Olap, SGC, Olap + SGC) and they received an intraperitoneal injection of Olaparib (Ola, 50 mg/kg), SGC0946(SGC, 50 mg/kg), Ola (50 mg/kg) + SGC (SGC, 50 mg/kg) or PBS three times a week. Three weeks post-injection, the mice were sacrificed, and mouse body weights and tumor weights were quantified. Tumors from each group are shown. **F**–**G**. The tumor volume curve (**F**) and weight of each group (**G**) were shown. **H**. The difference in nude mice’s body weights of each group before and after administration. (Data are presented as the mean ± SD, ns, *p* > 0.05; *p < 0.05, *n* = 5). **I**–**K**. The synergistic effects of SGC0946 and Olaparib on the viability of the indicated PDOs. Organoids were exposed for 5 days to combine treatments with suboptimal doses of SGC0946 (10 and 20 µM), and Olaparib (5 and 10 µM) (**I**). CI values less than 1, which suggest synergism, were calculated for drug combinations relative to the individual drugs and are indicated in the above graphs (**J**–**K**) (Data is presented as the mean ± SD, *** < 0.001, *n* = 3). **L**. Schematic diagram of molecular mechanism. Working model of the role of DOT1L in PARPi resistance

As previous work has identified the functional role of DOT1L in PARPi resistance, we next explore the clinical role of inhibiting DOT1L in PARPi resistance in vitro and in vivo. The combination index (CI) and synergistic lethal effect of drug combinations were detected by the CCK8 cell proliferation assay and analyzed by SynergyFinder or Compusyn software. The results show that the combination of the DOT1L inhibitor SGC0946 and Olaparib or Niraparib can significantly inhibit the proliferation of R8 OlaR and SV3 OlaR cells. Moreover, the CI values of the drugs are significantly less than 1 (Fig. 6A-B, Fig. S7A-B), indicating that inhibition of DOT1L can significantly reverse PARPi resistance and has an obvious synergistic effect with PARPi too. The colony formation and flow cytometry assays were further conducted in R8 OlaR and SV3 OlaR cells following PARPi treatment. As shown in Fig. 6C, the colony formation ratio was dramatically decreased in the presence of co-treatment with DOT1Li and PARPi compared to DOT1Li or PARPi alone. Additionally, increased apoptosis was observed in the combination therapy (Fig. 6D, Fig. S7C).

Next, we turned to investigate whether DOT1Li could overcome Olaparib resistance in the mouse xenograft model. BALB/c nude mice were subcutaneously injected with R8 OlaR cells. Approximately one week later, the mice were randomly assigned to four groups and intraperitoneally injected with a reference solvent: 50 mg/kg Olaparib, 50 mg/kg SGC0946, and Olaparib (50 mg/kg) + SGC0946 (50 mg/kg) three times a week for three weeks. The tumor volume and weight were significantly lower in the combination groups than in the Olaparib or SGC0946 single drug groups (Fig. 6E-G). The body weights of the nude mice in each group remained unchanged before and after administration (Fig. 6H). This demonstrates the antitumor role of DOT1Li in PARPi-resistant tumors in vivo. PDO model results also indicated that SGC0946 could enhance the effect of PARPi (Olaparib and Niraparib), and the synergistic effect of the SGC0946/Olaparib combination was strong in low concentration conditions (Fig. 6I-K, Fig. S7D-E). Furthermore, the DOT1Li (SGC0946) treatment abrogated the upregulation of PLCG2 and ABCB1, which was evident in PARPi-treated cells alone (Fig. SF-G). These results provide further evidence that inhibition of DOT1L could be a potential therapy for PARPi-resistant tumors.

## Discussion

The aberrant expression of DOT1L is thought to be correlated with an increased proliferative rate, augmented metastatic capacity, and unfavorable outcomes in several tumor types [42, 43], even though the knockdown and overexpression of DOT1L alone have no apparent effect on colony formation or apoptotic assays in our present results. This may depend on the cell lines adopted and the intrinsic status and activity of the cells when tested. In fact, previous literature showed that DOT1L appears to influence cancer cell migration, invasion, and metastasis more prominently [28, 44, 45], agreeing with our results depicted in Fig. S2I, that the DOT1L expression is correlated with OV cancer metastasis. In this study, we demonstrated that DOT1L is an independent prognosticator for patients with ovarian high-grade serous carcinoma, exhibiting cellular functions of a tumor promoter. This may influence the development of PARPi resistance and the progression of OC cells through regulation of gene expression. Our results suggest that DOT1L may serve as a novel biomarker for tumor development and a potential target for PARPi diagnosis and drug resistance in OC (Fig. 6L).

Dysregulated epigenetic regulators have been demonstrated to play a vital role in the development of chemotherapeutic resistance. Here, the gene profiles in PARPi-resistant cells compared to the original cells show a significant change among epigenetic regulator genes, including DOT1L and HMGA2. HMGA2 expression correlates with the level of malignancy directly, and is linked to enhanced metastatic potential and poor clinical outcomes in different cancers. Furthermore, it has been reported that HMGA2 acts as a functional antagonist of PARP1 inhibitors in breast cancer cell lines [36]. In this research, we identified that DOT1L expression levels were positively associated with PARPi resistance. Genetic and pharmacologic disruption of DOT1L could re-sensitize a subset of resistant models to PARPi. These findings further highlight the potential clinical utility of targeting epigenetic regulators in the context of drug resistance.

In the current study, CUT&Tag was employed to investigate the specific transcriptional mechanisms by which DOT1L contributes to PARPi resistance. This was done by collecting cells following Olaparib treatment, which may have led to the global difference being less evident. The spike-in (Drosophila epigenome incorporated) was utilized for ChIP-Seq normalization, which could facilitate the discernment of alterations in histone mark signal across the samples [46], and is gradually receiving attention and is being mentioned in conjunction with CUT&Tag in recent publications. Although the candidate targets were chosen after a conjoint analysis with RNA-seq and verified at both cellular and molecular levels, we would attach great importance to spike-in for subsequent related work aimed at exploring more prominent biological markers.

In recent years, PARPi has demonstrated activity in non-BRCA mutated tumors, presumably through induction of PARP1-DNA trapping [47]. An abundance of knowledge has been built around resistance mechanisms in BRCA-mutated tumors [11]. However, parallel understanding in non-BRCA mutated settings remains insufficient. In the current study, we reported that DOT1L promoted PARPi resistance in BRCA wild-type OC cells. Our findings indicate that DOT1L plays a role in regulating ABC transport genes and PLCG2 transcription, which eventually contributes to drug resistance. Knockdown of DOT1L dramatically enhanced the sensitivity of OC cells to PARPi, while overexpression of DOT1L resulted in increased resistance of OC cells to PARPi. Thus, targeted deletion of DOT1L may have potential clinical implications in overcoming PARPi resistance, regardless of the patients’ BRCA1/2 or HR-mediated DNA repair status. In addition, the upregulation of DOT1L following PARPi treatment was not observed in cisplatin-resistant OC cells (Fig. S1K-M), suggesting that DOT1L, as a key regulator of drug resistance, only contributed to acquired resistance to PARPi.

Inhibition of PARP activity is synthetically lethal in cells with HR deficiency. One of the main mechanisms involved in PARPi resistance is HR restoration [13]. In this paper, we elaborate on the mechanisms of DOT1L that contribute to PARPi resistance through gene profile. Indeed, in addition to gene transcription regulation, some literature reports indicate that DOT1L is involved in the DNA damage repair (DDR) process, such as 53BP1 recruitment to double-strand breaks (DSBs), which is dependent on DOT1L-catalyzed H3K79me2 in G1/G2 in U2OS [48]. In addition, DOT1L has been reported to regulate the phosphorylation of the variant histone H2AX (γH2AX) to participate in the early DNA damage response [49]. This suggests that DOT1L may contribute to PARPi resistance by promoting DDR as well. The specific mechanism by which this occurs requires further investigation.

Although the DNA repair effects of the PARP inhibitors are likely to represent a significant component of their mode of action, the actions of PARP inhibitors are likely to be pleiotropic and extend well beyond DNA repair, including gene regulation [37, 50]. In this regard, more evidence indicates that PARPi may decrease the apoptotic threshold in cotreatments with chemotherapies by regulating the expression of tumor-related genes [50, 51]. Here, our findings indicate that PARP1-DNA trapping, accompanied by PARPi treatment, results in increased transcription of DOT1L and contributes to PARPi-acquired resistance. Co-treatment with DOT1Li has the potential to enhance the sensitivity to PARPi and reverse the resistance to PARPi. Thus, our study further suggests that when evaluating the molecular mechanisms of PARP inhibitors, gene expression programs should be considered in addition to DNA repair outcomes. This may be especially pertinent under conditions of chronic administration, in which changes in gene expression over the long term may have long-lasting effects on the cell and organism.

Except for DNA damage repair, PARP1 is also involved in gene expression regulation. It is evident that PARP1 can regulate gene expression through a variety of mechanisms, including its function as a coregulator for DNA-binding transcription factors, a modulator of chromatin, and a regulator of DNA methylation [37, 38, 50]. Furthermore, its role in gene expression regulation is multifaceted. For instance, PARP1 plays a pivotal role in facilitating transcription through nucleosomes by Pol II, and its catalytic activity is essential for this process [52]. Other studies have reported that PARP1 impairs the transcription of PD-L1 by poly(ADP-ribosyl)ating STAT3, thereby reducing the transcriptional activity of STAT3 [53]. In addition, PARP1 protein has been shown to regulate SNAI2 transcription by influencing the chromatin accessibility around SNAI2 promoters. Talazoparib treatment has been demonstrated to drive SNAI2 transcription and confer drug resistance [54]. Here, our findings indicate that PARPi treatment facilitates DOT1L transcription and drug resistance. This is evidenced by the observation that PARP1-DNA trapping increased binding of PARP1 on the DOT1L promoter and that PARP1 could increase STAT3 binding to the DOT1L promoter. Moreover, PARPi treatment could enhance the transcriptional activity of STAT3 [53], which in turn further promotes DOT1L gene expression. Consequently, further research is currently being conducted to better elucidate the structural characteristics of PARP1, to ascertain whether PARP1-induced chromatin accessibility differs between specific gene regions, and to identify the role of enzyme activity in this process. In conclusion, our study provides further evidence that PARP1-mediated gene regulation contributes to therapy outcomes.

## Conclusions

In conclusion, our findings identify DOT1L as a potential driver of PARPi resistance and discover a novel crosstalk between DOT1L and PARP1, in which PARP1 induces upregulation of DOT1L independent of its catalytic activity. Since PARPi has been used to treat cancer patients, this newly identified activity raises the question of whether PARPi-induced DOT1L activity increases tumor progression and drug resistance, which could attenuate the therapeutic efficacy of PARPi. Our research expanded the explanation of PARPi-acquired resistance and provided an experimental basis for overcoming PARPi resistance clinically. Inhibition of DOT1L combined with PARPi may have broad prospects in the clinical therapy of ovarian cancer.

### Electronic supplementary material

Below is the link to the electronic supplementary material.

Supplementary Material 1

Supplementary Material 2

## Data Availability

The CUT&Tag and RNA-seq data of our local samples have been deposited in the Genome Sequence Archive (GSA) in the National Genomics Data Center, China National Center for Bioinformation/Beijing Institute of Genomics, Chinese Academy of Sciences (https://ngdc. cncb.ac.cn/gsa-human), Chinese Academy of Sciences, under accession numbers HRA007199, HRA007200 and HRA007266.

## Abbreviations

PARPi: Poly (ADP-ribose) polymerase inhibitor (PARPi)
OC: ovarian carcinoma
ChIP: chromatin immunoprecipitation
CUT&Tag assays: Cleavage Under Targets and Tagmentation assays
CDXs: cell line-derived xenograft mouse models
PDOs: patient-derived organoids
ABC transporter: ATP-binding cassette (ABC) transporter
PLCG2: Phospholipase C, gamma 2
