# Supplementary material for: PARP1-DOT1L transcription axis drives acquired resistance to PARP inhibitor in ovarian cancer
Source: Mol Cancer. 2024 May 22;23:111. doi: 10.1186/s12943-024-02025-8 (PMC11110363; doi:10.1186/s12943-024-02025-8)
Supplement: Supplementary file 1 — Supplementary Material 1 [file 12943_2024_2025_MOESM1_ESM.docx]

**Figure S1. Upregulated DOT1L expression correlates with PARPi resistance in OC. Related to Figure 1**


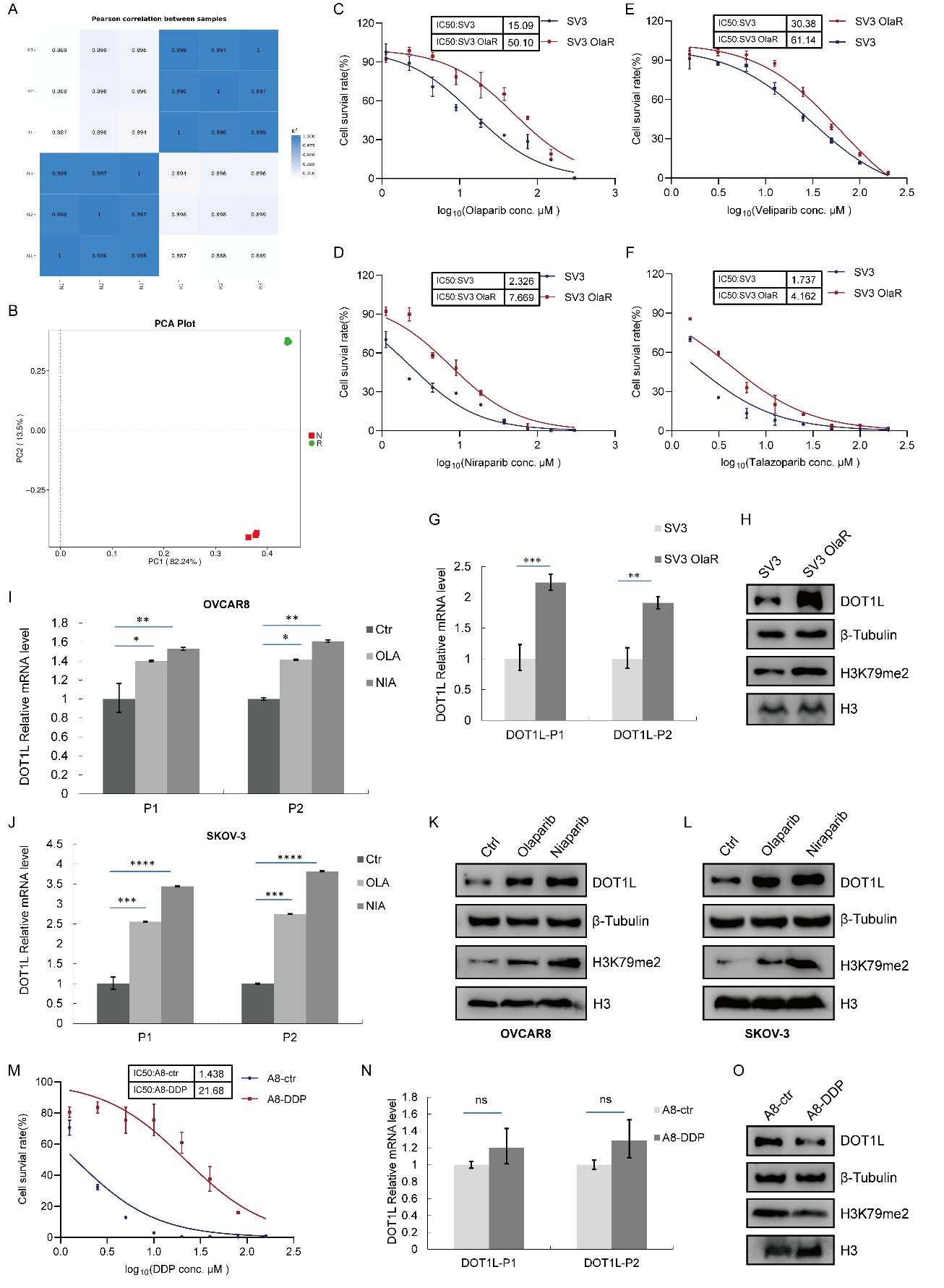


A. Pearson’s correlation analysis showing a high degree of differentiation between the mRNA expression profiles of the R8 OlaR (R) (n = 3) and R8 (N) (n = 3) samples. B. Principal component analysis (PCA) of the transcriptomic data segregated the samples into two groups. C. Olaparib IC50 curves of parent SKOV-3 cells and cells with acquired resistance to Olaparib. D. Niraparib IC50 curves of parent SKOV-3 cells and cells with acquired resistance to Olaparib. E. Veliparib IC50 curves of parent SKOV-3 cells and cells with acquired resistance to Olaparib. F. Talazoparib IC50 curves of parent SKOV-3 cells and cells with acquired resistance to Olaparib. G. DOT1L mRNA levels in SV3 OlaR and its original parent SKOV-3 cells were analyzed by RT-qPCR. (Data is presented as the mean ± SD, and two independent sets of DOT1L primers were used. **p < 0.01, ***p < 0.001, n = 3). H. Olaparib-resistant SKOV-3 (SV3 Ola-R) and its original parent SKOV-3 (SV3) cells were collected, and western blotting was conducted to detect with the indicated antibodies. I-J. DOT1L mRNA levels in OVCAR8 (I) and SKOV-3 (J) cells with PARPi treatment (Olaparib, 10μm, 36h; Niraparib, 5μm, 36h) (Data is presented as the mean ± SD, *p < 0.05, **p < 0.01, ***p < 0.001; ****p < 0.0001, n = 3). K-L. DOT1L protein expression levels in OVCAR8 (K) and SKOV-3 (L) cells with PARPi treatment (Olaparib, 10μm, 36h; Niraparib, 5μm, 36h). M. IC50 of A2780-cispatin resistant (A8-DDP) and its original parent A2780 (A8-ctr) detected by CCK8 assay. N-O. DOT1L mRNA levels (M) and protein expression (O) in A8-DDP and A8-ctr cells.

**Figure S2. Clinical correlation analysis of DOT1L expression in OC. Related to Figure 1**


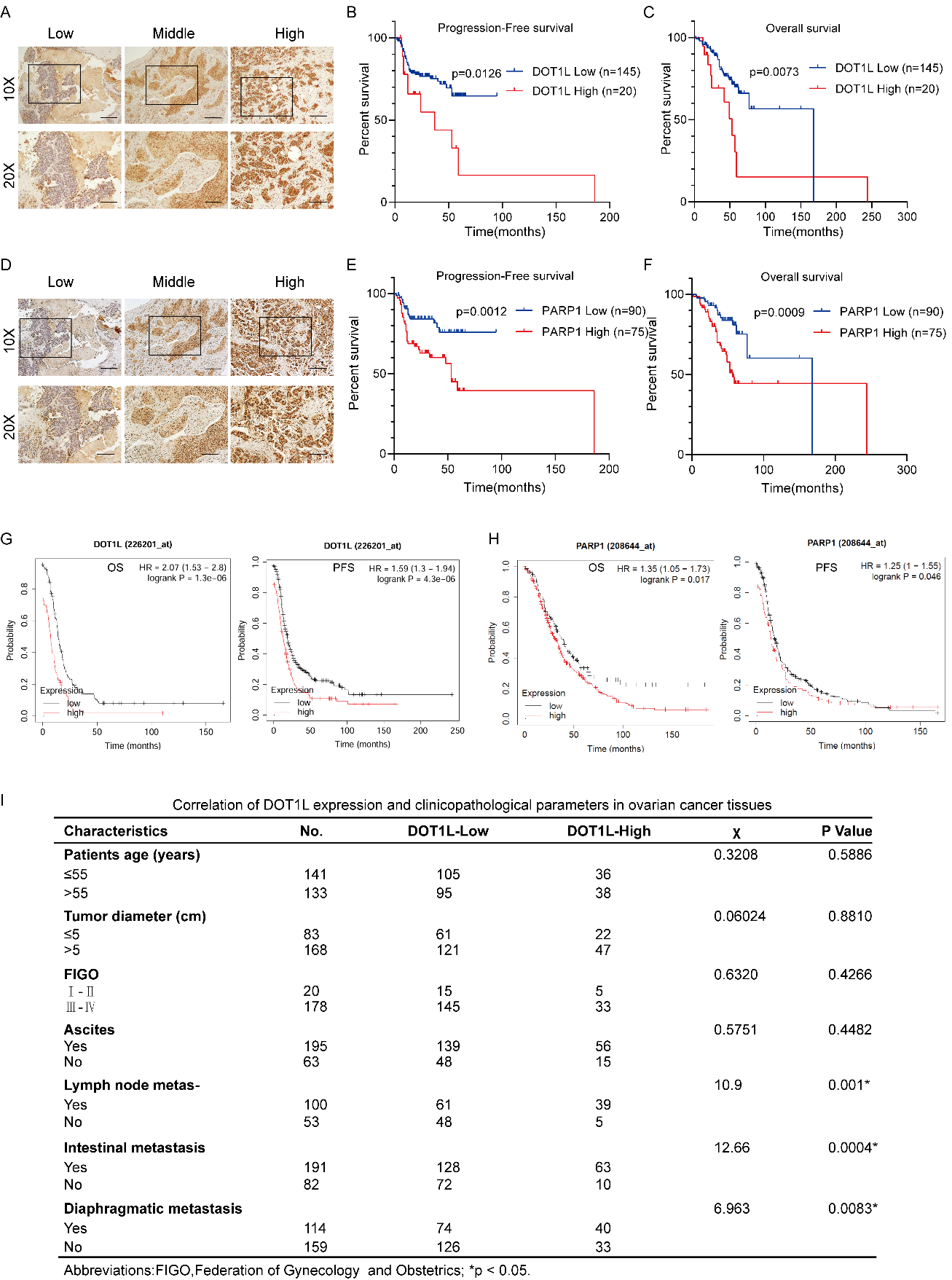


A. Representative images of IHC staining of DOT1L at different expression levels in OC TMAs. Scale bars: 200 μm (upper); 100 μm (lower). B-C. Progression-free survival (PFS) (B) and overall survival (OS) (C) curves of OC patients with high or low DOT1L levels in OC TMAs. The log-rank test was used to compare the survival curves between groups. D. Representative images of IHC staining of different PARP1 expression levels in OC TMAs. Scale bars: 200 μm (upper); 100 μm (lower). E-F. Progression-free survival (PFS) (E) and overall survival (OS) (F) curves of OC patients with high or low PARP1 levels in OC TMAs. The log-rank test was used to compare the survival curves between groups. G-H. OS and DFS of OC patients with respect to DOT1L (G) and PARP1 (H) were analyzed by the Kaplan-Meier Plotter. I. Correlation between DOT1L expression and clinicopathological parameters in ovarian cancer tissues.

**Figure S3. DOT1L regulates OC sensitivity to Olaparib and contributes to PARPi resistance. Related to Figure 2**


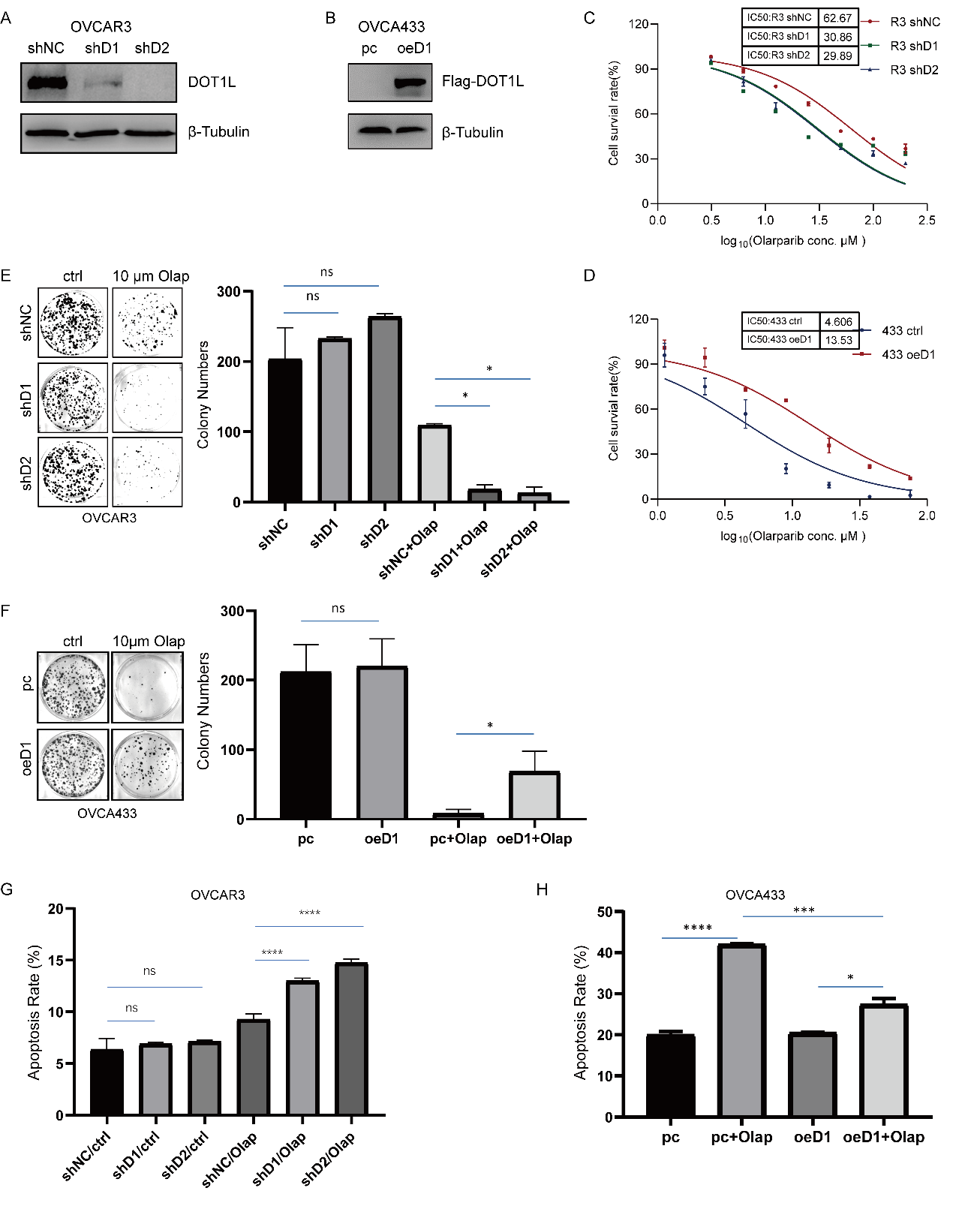


A. PLKO.1, DOT1L shRNA (shDOT1L) plasmids were stably transfected into OVCAR3 cells, Western blot was used to determine DOT1L protein levels. B. PCMV, and PCMV DOT1L plasmids were stably transfected into OVCA433 cells. Western blot was used to determine DOT1L protein levels. C. The CCK8 assay was performed to detect cell viability in OVCAR3 cells treated with Olaparib (Olap) for 96 h. D. The CCK8 assay was performed to detect cell viability in OVCA433 cells treated with Olaparib (Olap) for 96 h. E. A clonogenic assay was conducted to assess the colony formation efficiency of OVCAR3 cells in the presence of Olaparib for 7–14 days (left) and quantity the number of clones (right). F. A clonogenic assay was conducted to assess the colony formation efficiency of OVCA433 cells in the presence of Olaparib for 7–14 days (left) and quantify the number of clones (right). G. A flow cytometry assay was performed to detect cell apoptosis in OVCAR3 cells treated with Olaparib (10 μM) for 48 h. H. A flow cytometry assay was performed to detect cell apoptosis in OVCA433 cells treated with Olaparib (10 μM) for 48 h. (Data is presented as the mean ± SD; ns, p > 0.05; *p< 0.05, **p < 0.01, ***p < 0.001; ****p < 0.0001, n = 3).

**Figure S4. PARP1-mediated transcription regulation directly influences DOT1L expression. Related to Figure 3**


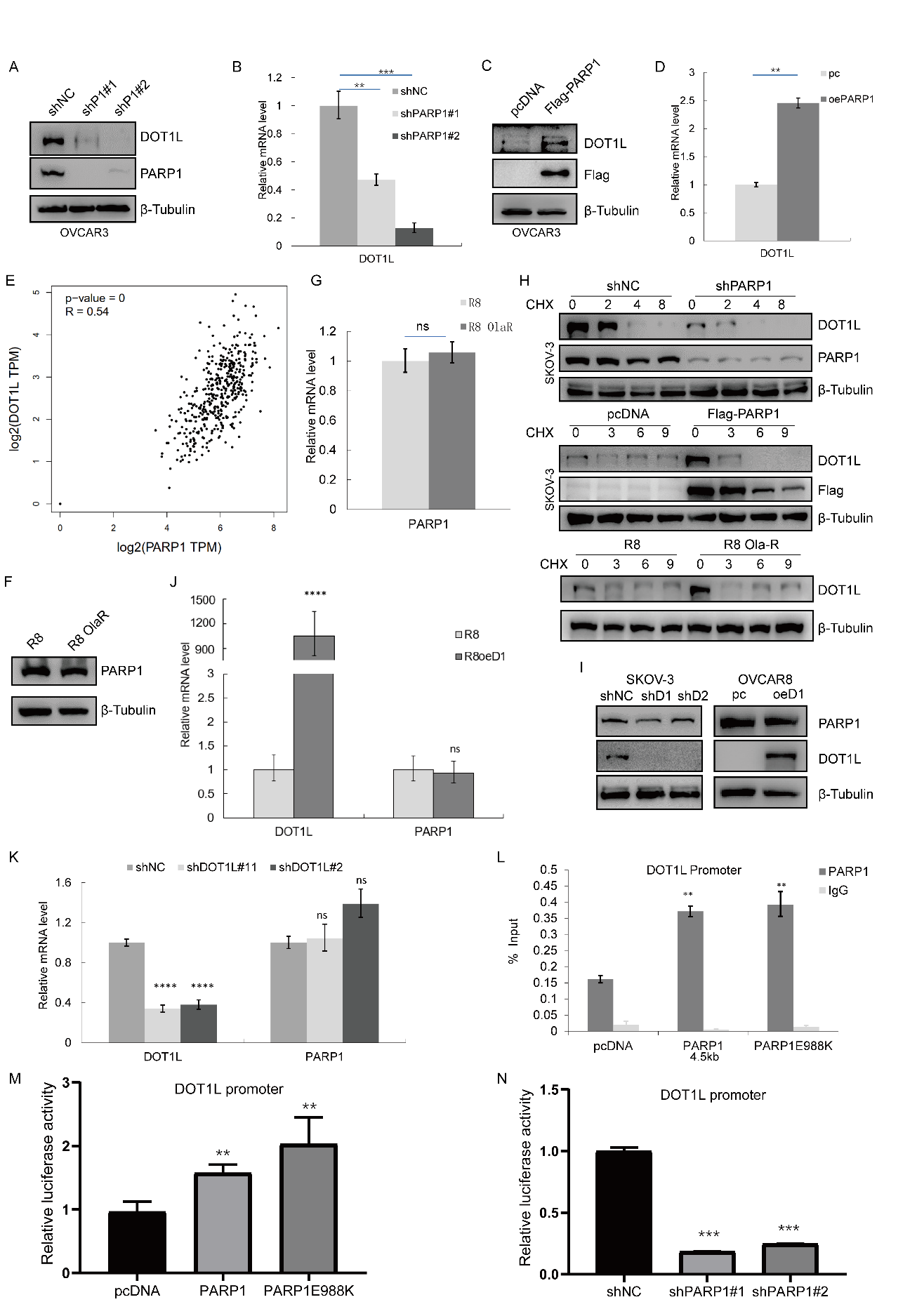


A. PARP1 stably knockdown OVCAR3 cells were constructed, and whole cell lysates (WCL) were extracted and analyzed by western blotting with the indicated antibodies. B. RT-qPCR was used to determine the DOT1L and PARP1 mRNA levels in PARP1 knockdown OVCAR3 cells. C. Whole cell lysates (WCL) were extracted from PARP1 stably overexpressed OVCAR3 cells and analyzed by western blotting with the indicated antibodies. D. RT-qPCR was used to determine the DOT1L and PARP1 mRNA levels in PARP1 overexpressed OVCAR3 cells. E. The positive correlation between DOT1L and PARP1 was analyzed by GEPIA. F-G. OVCAR8 and OVCAR8 OlaR cells were collected, and western blotting and RT-qPCR were performed to detect PARP1 protein expression (F) and mRNA (G) levels. H. SKOV-3 PARP1 knockdown (upper), Flag-PARP1 overexpressed (middle), or OVCAR8 Olaparib resistant cells (lower) were treated with 50 μg/ml of cycloheximide (CHX), and the half-life period of DOT1L protein was determined by western blotting. I. Western blotting was performed in DOT1L knockdown SKOV-3 and DOT1L overexpressed OVCAR8 cells to determine PARP1 protein expression levels. J-K. RT-qPCR was performed in DOT1L overexpressed OVCAR8 (J) and DOT1L knockdown SKOV-3 (K) cells to determine PARP1 mRNA expression levels. L. A PARP1-ChIP assay was performed in pcDNA-, PARP1(WT)-, or PARP1(E988K)-transfected SKOV-3 cells to examine PARP1 occupancy at DOT1L. M. OVCAR3 cells were transfected with the DOT1L promoter report gene, together with control pcDNA, wild Flag-PARP1, and mutant Flag-PARP1 (E988K) as indicated. The luciferase activity was measured 36 hours after transfection. N. The luciferase reporter assay was performed in PARP1 stably knockdown OVCAR3 cells.

**Fig S5. DOT1L facilitates PARPi resistance via H3K79 methylation. Related to Fig. 4**


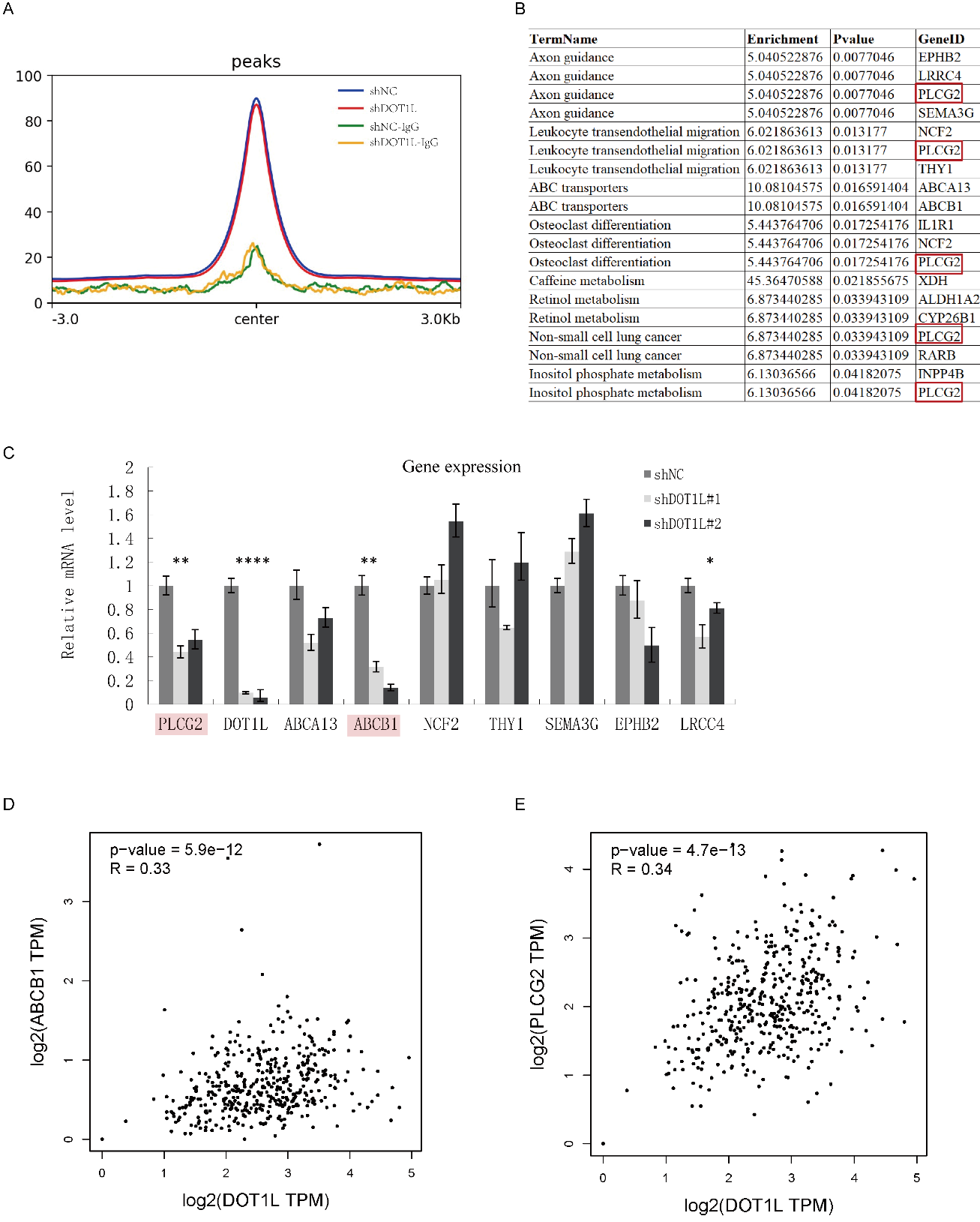


A. Normalized read distribution profiles of H3K79me2 CUT&Tag spanning 3 kb of gene bodies in control (shNC) and DOT1L knockdown (shDOT1L) SKOV-3 cells treated with Olaparib 10μM for 48h. IgG was used as the negative control. B. The KEEP analysis revealed the significantly enriched items based on the H3K79me2 signature. C. RT-qPCR performed in DOT1L knockdown SKOV-3 cells shows the mRNA expression levels of candidate genes (B). D-E. The positive correlation between ABCB1 (D) or PLCG2 (E) and DOT1L was analyzed by GEPIA tool.

**Figure S5. PARP1-DOT1L-PLCG2/ABCB1 axis contributes to PARPi resistance**


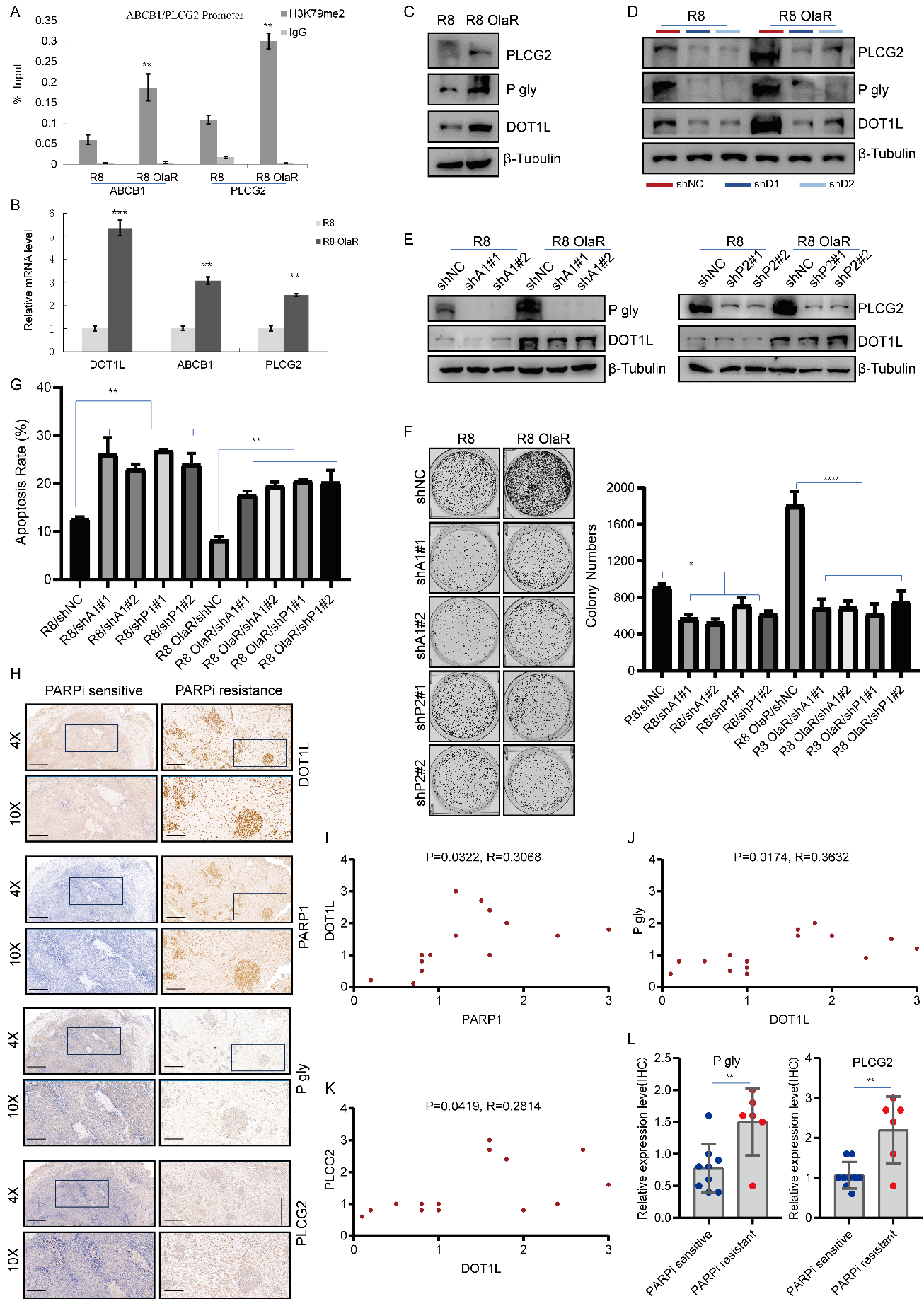


A. H3K79me2-ChIP assay was performed with OVCAR8 Olaparib-resistant and parent OVCAR8 cell lines to determine H3K79me2 occupancy at PLCG2 and ABCB1. B. PLCG2 and ABCB1 mRNA levels were determined in R8 OlaR and parent OVCAR8 cells by RT-qPCR. C. Western blotting was performed in Olaparib-resistant OVCAR8 and parent OVCAR8 cell lines to examine PLCG2 and ABCB1 (P-gly) protein expression levels. D. Western blotting was performed in R8 OlaR and parent OVCAR8 cells which were transfected with shNC and DOT1L shRNA respectively with the indicated antibodies. E. R8 OlaR and parent OVCAR8 cells were transfected with shNC, ABCB1 shRNA and PLCG2 shRNA. After 72 hours of transfection, cells were collected and analyzed by western blotting with the indicated antibodies. F-G. Colony formation (F) and cell apoptosis assay (G) were performed in R8 OlaR and parent OVCAR8 stably transfected cell lines. H. Immunohistochemistry (IHC) staining of DOT1L, PARP1, ABCB1 (P-gly), and PLCG2 in PARP inhibitor-resistant human ovarian carcinomas (OC) tissues and sensitive tissues. Representative images are shown. Scale bars: 400 μm (upper); 160 μm (lower). I-K. Correlation analysis between PARP1 and DOT1L(I), DOT1L and P-gly (J), and DOT1L and PLCG2 (K) were analyzed. L. Quantification of P-gly (right) and PLCG2 (left) expression in PARP inhibitor-resistant OC tissues (n = 6) and sensitive tissues (n = 9), **p < 0.01.


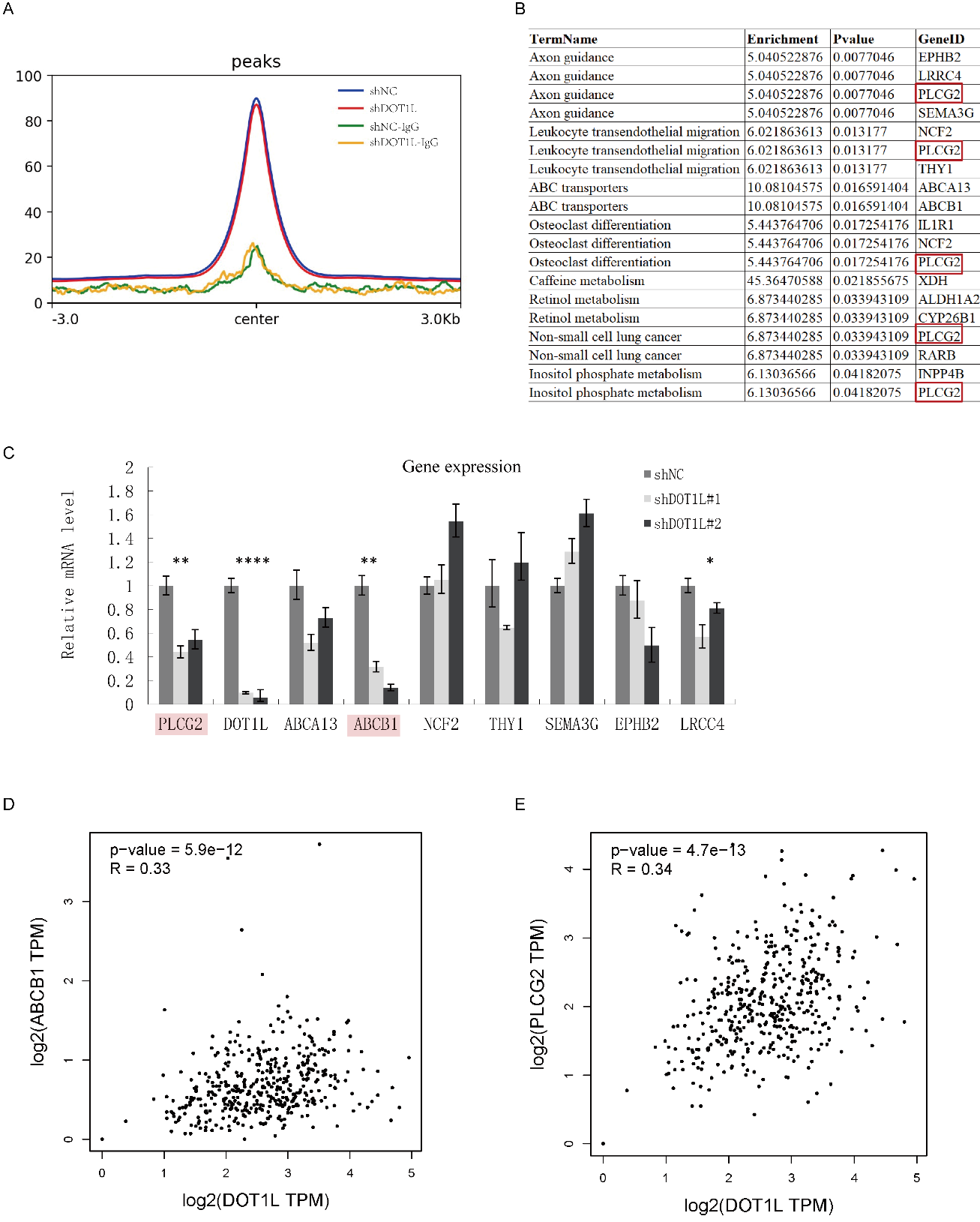


**Figure S6. PARP1-DOT1L-PLCG2/ABCB1 axis contributes to PARPi resistance. Related to Figure 5**


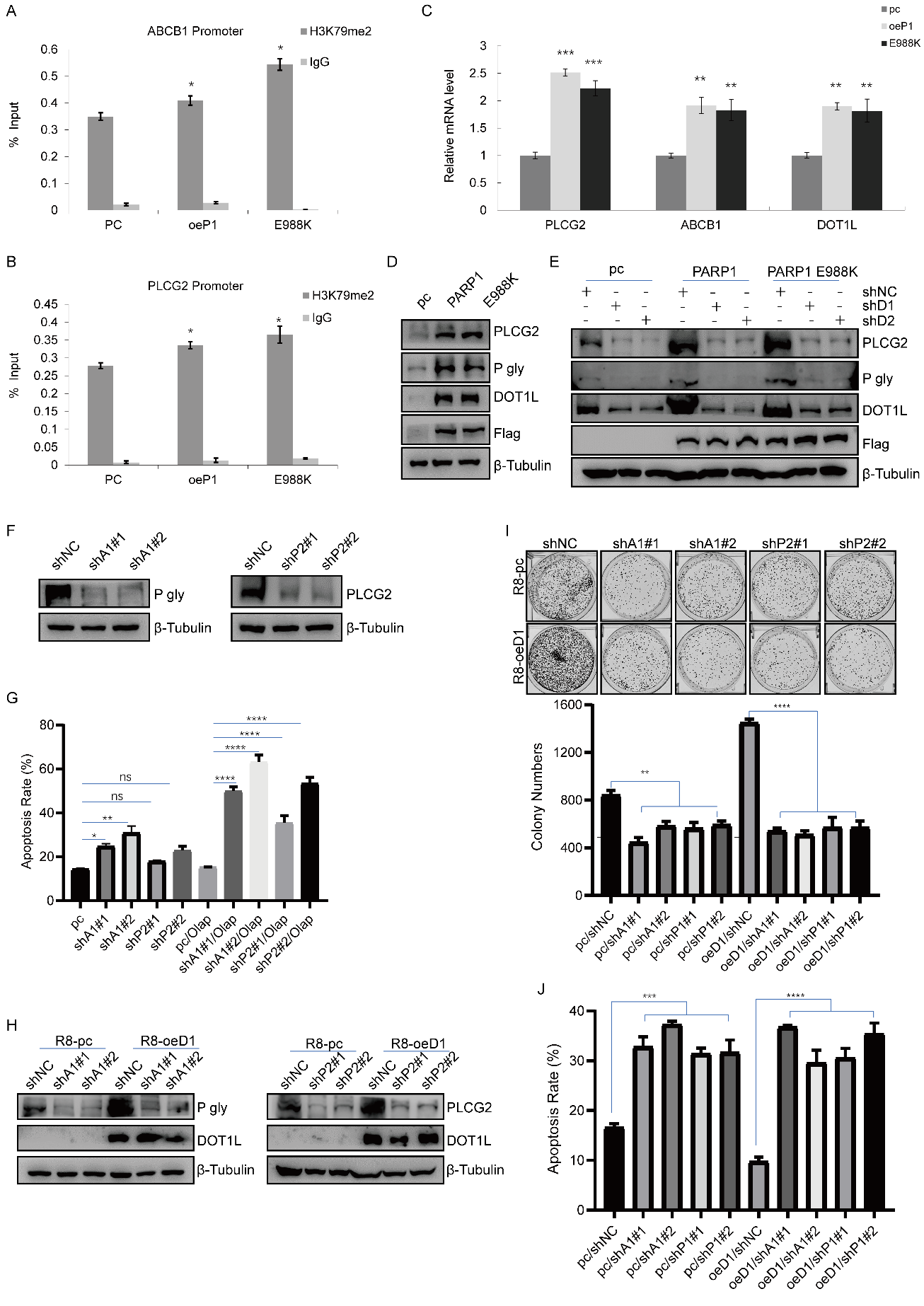


A-B. H3K79me2-ChIP assay was performed in pcDNA-, PARP1(WT)-, or PARP1 E988K-transfected SKOV-3 cells to examine H3K79me2 occupancy at ABCB1(A) and PLCG2(B). C. RT-qPCR performed in pcDNA-, PARP1(WT)-, or PARP1 E988K-transfected SKOV- 3 cells to examine PLCG2 and ABCB1 mRNA expression levels. D. SKOV-3 cells transfected with pcDNA, PARP1(WT), or PARP1 E988K plasmids. Whole cell lysates (WCL) were collected and conducted with western blotting with the indicated antibodies. E. pcDNA-, wild Flag-PARP1-, and mutant Flag-PARP1 (E988K)- transfected SKOV-3 cells were transfected with shRNA and DOT1L shRNA and subjected with western blotting to detect indicated protein expression levels. F. SKOV-3 cells were transfected with shNC, shABCB1, or shPLCG2. Whole cell lysates were collected and analyzed by western blotting to determine ABCB1 (P-gly) and PLCG2 expression levels. G. Cell apoptosis was performed in (F) to examine the sensitivity of OV cells to PARPi with PLCG2 or ABCB1 interference. H. shNC-, shPLCG2-, and shABCB1 were transfected to DOT1L stably overexpressed OVCAR8 and its original cells. The cell lysates were collected and analyzed by western blotting with the indicated antibodies. I-J. colony formation (I) and cell apoptosis assay (J) were performed in (H).

**Figure S7. Targeted inhibition of DOT1L sensitizes OC to PARPi in vitro and in vivo. Related to Figure 6**


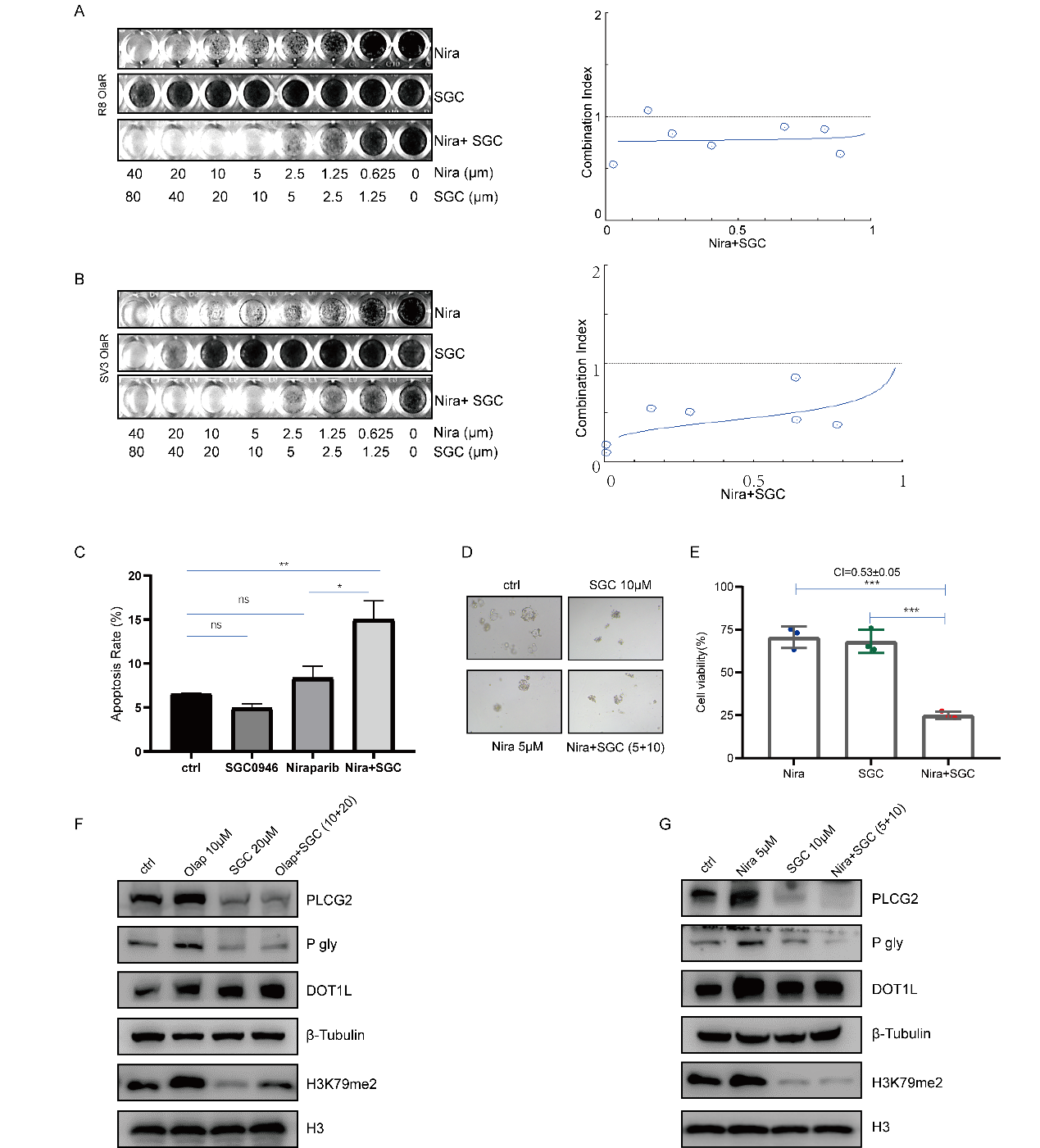


A-B. CCK8 assays were performed in R8 OlaR(A) and SV3 OlaR (B) cells to detect the synergistic effect between DOT1L inhibitor SGC0936 (SGC) and PARP inhibitor Niraparib (Nira) (left). Compusyn software was used to calculate the synergy index (CI) of DOT1L inhibitor combined with PARP inhibitor(right): CI value less than 1 indicates a synergistic effect, a CI value greater than 1 indicates an antagonistic effect, and a CI value equal to 1 indicates a superposition effect. C. A flow cytometry assay was performed to detect cell apoptosis in SV3 OlaR cells treated with Niraparib (5μM), SGC0946 (10μM), or Niraparib (5μM) + SGC0946 (10μM) for 48 h. D-E. Synergistic effects of SGC0946 and Niraparib on the viability of the indicated PDOs. Organoids were exposed for 5 days to combined treatments with suboptimal doses of SGC0946 (10 μM), Niraparib (5 μM) (D). CI values less than 1, which suggest synergism, were calculated for drug combinations relative to the individual drugs and are indicated above the graphs (E). (Data are presented as the mean ± SD, **p < 0.01, *** < 0.001, n = 3). F. Western blots were performed in PDOs treated with SGC0946 or Olaparib with the indicated antibodies. G. Western blots were performed in PDOs treated with SGC0946 or Niraparib with the indicated antibodies.
