# Supplementary material for: PARP1-DOT1L transcription axis drives acquired resistance to PARP inhibitor in ovarian cancer
Source: Mol Cancer. 2024 May 22;23:111. doi: 10.1186/s12943-024-02025-8 (PMC11110363; doi:10.1186/s12943-024-02025-8)
Supplement: Supplementary file 2 — Supplementary Material 2 [file 12943_2024_2025_MOESM2_ESM.docx]

Supplementary Table 1.

**ChIP and Real-Time PCR Primers**

| Target Gene | Forward primer | Reverse primer |
| --- | --- | --- |
| ChIP_DOT1L-1 | GGACCGTGACTCTTATGGGG | GGTTCGAATCCCGTCCCAG |
| ChIP_DOT1L-2 | GAGCAGTTCGGAAGGGGTTT | CATGCGGGGCTTGAGAAAAG |
| ChIP_DOT1L-3 | ATCCCTGCATTGAGCCCTTC | TTTGAACCTTCCAGGGCCAG |
| ChIP_DOT1L-4 | GGGGGTAGCCCTGCTTTTAT | GCAGCAAGCCTGGATCTTG |
| ChIP_PLCG2-1 | TTCATGCAACACACGAACGG | GTCAGGAGCAAAGAACCCCA |
| ChIP_PLCG2-2 | GAGGCAAAGGAATTGCCCCT | TCCCAACTGGCGTGTATAGC |
| ChIP_PLCG2-3 | GTCTAGGTGCCCCTGAAACT | ACCCCAGACAATTGATGTCCC |
| ChIP_ABCB1-1 | TGCTTTGGAGCCATAGTCATGT | TAAGGCAGGCAGGCTTGAAA |
| ChIP_ABCB1-2 | AGACGTCCTACACCTTAGCA | CTAAGGCAGGCAGGCTTGAA |
| ChIP_ABCB1-3 | AGGTCTTCCCAGTAACCTACCA | CATGGCTTAGGGATTGGGGT |
| RT_Actin | CATGTACGTTGCTATCCAGGC | CTCCTTAATGTCACGCACGAT |
| RT_DOT1L-1 | CTGCCGGTCTACGATAAACATC | AGCTTGAGATCCGGGATTTCT |
| RT_DOT1L-2 | AATCCCGGATCTCAAGCTCG | GCACGGTTGTACTTGTCGC |
| RT_PARP1-1 | ACCTCATCAAGATGATCTTT | TTGAGAGATCCAGGATCTGA |
| RT_PARP1-2 | CGGAGTCTTCGGATAAGCTCT | TTTCCATCAAACATGGGCGAC |
| RT_ABCB1 | TTGCTGCTTACATTCAGGTTTCA | AGCCTATCTCCTGTCGCATTA |
| RT_PLCG2 | TCCACCACGGTCAATGTAGAT | CCCTGGGCGGATTTCTTTTAT |
| RT_ABCA13 | CAGCTATGAAGGGTCAATGGAG | TGGATCGTTCTACCCAAAGTCT |
| RT_NCF2 | CCCACTCCCGGATTTGCTTC | GTCTCGGTTAATGCTTCTGGTAA |
| RT_THY1 | ATCGCTCTCCTGCTAACAGTC | CTCGTACTGGATGGGTGAACT |
| RT_SEMA3G | CTCTGCGTTCCGACTCTGAC | CTGACAGTGACATGGTTCGAG |
| RT_EPHB2 | AGAAACGCTAATGGACTCCACT | GTGCGGATCGTGTTCATGTT |
| RT_LRCC4 | CTCCCGTTCGTCTACCTCAC | GGTGTTCGAGGGAATACCCTG |

**shRNA sequences**

| Target Gene | Sequence |
| --- | --- |
| DOT1L-1 | CCGAGAAGCUCAACAACUA |
| DOT1L-2 | GCUCGCUAUGGAGAAUUAC |
| PARP1-1 | GGACCAAGTGTATGGTCAAGA |
| PARP1-2 | GCCCTTTAAGCAGCTTCATAA |
| ABCB1-1 | GGAUGAAAGUAUACCUCCA |
| ABCB1-2 | CUAAUAGAAGUGAUAUCAA |
| PLCG2-1 | GUACAUGUGGGAUUCCAUU |
| PLCG2-2 | CUGAGAAAGCAGAUAUAUU |
